# CODEX: a normalization and copy number variation detection method for whole exome sequencing

**DOI:** 10.1093/nar/gku1363

**Published:** 2015-01-23

**Authors:** Yuchao Jiang, Derek A. Oldridge, Sharon J. Diskin, Nancy R. Zhang

**Affiliations:** 1 Genomics and Computational Biology Graduate Program, Perelman School of Medicine, University of Pennsylvania, Philadelphia, PA 19104, USA; 2 Medical Scientist Training Program, Perelman School of Medicine, University of Pennsylvania, Philadelphia, PA 19104, USA; 3 Division of Oncology and Center for Childhood Cancer Research, The Children's Hospital of Philadelphia, Philadelphia, PA 19104, USA; 4 Department of Pediatrics, Perelman School of Medicine, University of Pennsylvania, Philadelphia, PA 19104, USA; 5 Abramson Family Cancer Research Institute, Perelman School of Medicine, University of Pennsylvania, Philadelphia, PA 19104, USA; 6 Department of Statistics, The Wharton School, University of Pennsylvania, Philadelphia, PA 19104, USA

## Abstract

High-throughput sequencing of DNA coding regions has become a common way of assaying genomic variation in the study of human diseases. Copy number variation (CNV) is an important type of genomic variation, but detecting and characterizing CNV from exome sequencing is challenging due to the high level of biases and artifacts. We propose CODEX, a normalization and CNV calling procedure for whole exome sequencing data. The Poisson latent factor model in CODEX includes terms that specifically remove biases due to GC content, exon capture and amplification efficiency, and latent systemic artifacts. CODEX also includes a Poisson likelihood-based recursive segmentation procedure that explicitly models the count-based exome sequencing data. CODEX is compared to existing methods on a population analysis of HapMap samples from the 1000 Genomes Project, and shown to be more accurate on three microarray-based validation data sets. We further evaluate performance on 222 neuroblastoma samples with matched normals and focus on a well-studied rare somatic CNV within the *ATRX* gene. We show that the cross-sample normalization procedure of CODEX removes more noise than normalizing the tumor against the matched normal and that the segmentation procedure performs well in detecting CNVs with nested structures.

## INTRODUCTION

Copy number variants (CNVs) are large insertions and deletions that lead to gains and losses of segments of chromosomes. CNVs are an important and abundant source of variation in the human genome (1–4). Like other types of genetic variation, some CNVs have been associated with diseases, such as neuroblastoma (5), autism (6) and Crohn's disease (7). Better understanding of the genetics of CNV-associated diseases requires accurate CNV detection. Traditional genome-wide approaches to detect CNVs make use of array comparative genome hybridization (CGH) or single nucleotide polymorphism (SNP) array data (8–10). The minimum detectable size and breakpoint resolution, which are correlated with the density of probes on the array, are limited. Paired-end Sanger sequencing, which is often used as the gold standard platform for CNV detection, has better resolution and accuracy but requires significant time and budget investment.

With the dramatic growth of sequencing capacity and the accompanying drop in cost, massively parallel next-generation sequencing (NGS) offers appealing platforms for CNV detection. Many current analysis methods are focused on whole genome sequencing (WGS), which allows for genome-wide CNV detection and finer breakpoint resolution than array-based approaches (11–15). Whole exome sequencing (WES), on the other hand, has been preferred as a cheaper, faster, but still effective alternative to WGS in large-scale studies, where the priority has been to identify disease-associated variants in coding regions (16–19).

**Figure 1.**
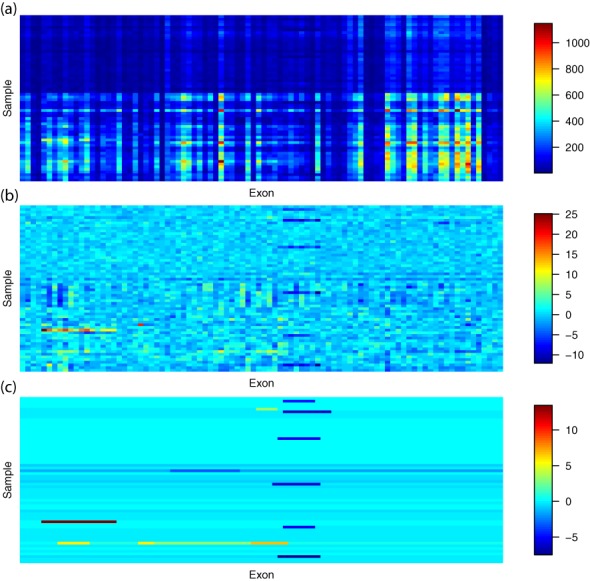
Heatmaps of raw, normalized and segmented WES read depth data from the TARGET Project. (a) Exon- and sample-wise biases and artifacts make the raw read depth data noisy and not directly reflect true copy number states. (b) CODEX's normalization procedures are performed. Heatmap of ${{\left( {Y - \widehat\lambda } \right)} / {\sqrt {\widehat\lambda } }}$ is shown and has a cleaner view of CNV signals. (c) CODEX's Poisson-likelihood-based segmentation procedure returns CNV calls.

Due to the biases and artifacts introduced during the exon targeting and amplification steps of WES, depth of coverage in WES data is heavily contaminated with experimental noise and thus does not accurately reflect the true copy number. Here, we present a novel normalization and CNV calling method, CODEX (COpy number Detection by EXome sequencing), to remove biases and artifacts in WES data and produce accurate CNV calls. As an example, in Figure 1a, we show heatmap of raw read depth matrix from the Therapeutically Applicable Research to Generate Effective Treatments (TARGET) Project (20) WES data set. The region contains a small deletion that is obscured by experimental noise. The second and third heatmaps show the coverage in this region after, respectively, CODEX's normalization and segmentation steps.

Several algorithms have been developed for copy number estimation with whole exome data in matched case/control settings by either directly using the matched normal (21–23) or building an optimized reference set (24,25) to control for artifacts. Other algorithms use singular value decomposition (SVD) to extract copy number signals from noisy coverage matrices by removing *K* latent factors that explain the most variance (26–28). This exploratory approach assumes continuous measurements with Gaussian noise, uses an arbitrary choice of *K* and does not specifically model known quantifiable biases, such as those due to GC content.

CODEX does not require matched normal controls, but relies on the availability of multiple samples processed using the same sequencing pipeline. Unlike current approaches, CODEX uses a Poisson log-linear model that is more suitable for discrete count data. The normalization model in CODEX includes terms that specifically remove biases due to GC content, exon length and capture and amplification efficiency, and latent systematic artifacts. We explore several different statistical approaches for choosing the number of latent factors, and discuss how one should set this crucial parameter wisely. The power of CODEX and SVD-based approaches are compared by *in silico* spike-in studies on the 1000 Genomes Project (29) WES data and show that CODEX offers higher power in detecting both common and rare CNVs. Also, on WES data from the 1000 Genomes Project paired with SNP array data from three previous cohort studies on the same HapMap samples (30–32), CODEX gives higher precision and recall for both rare and common CNV detection by WES data, as compared to existing methods. CODEX's normalization and segmentation accuracy is further evaluated through the analysis of the WES data of 222 neuroblastoma matched tumor/blood samples from the TARGET project (20), with a focus on the well-studied *ATRX* gene region (20,33,34). The cross-sample normalization procedure of CODEX, when applied to the matrix of tumor samples, is more effective in reducing noise than normalizing each tumor to its matched normal. The somatic deletions in the *ATRX* region have a nested structure, which CODEX was able to recover.

## MATERIALS AND METHODS

### Overview of analysis pipeline

**Figure 2.**
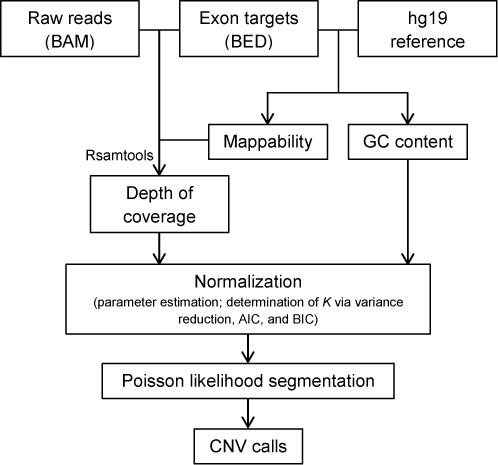
A flowchart outlining the procedures of CODEX in normalizing WES read depth and calling CNV. The first step is computing GC content, mappability and depth of coverage using Rsamtools with QC measures. The multi-sample normalization model by CODEX is then applied to remove biases and artifacts introduced by GC content, exon targeting and amplification efficiency and latent systemic artifacts. The Poisson likelihood-based segmentation algorithm gives final CNV calls with copy number estimates.

Figure 2 shows an overview of the analysis pipeline of CODEX. We start with mapped reads from BAM files (35) that are assembled, sorted and indexed by the same pipeline, and compute depth of coverage after a series of quality filtering based on mappability, exon size and a cutoff on minimum coverage (see details below). Then, we fit a normalization model based on a log-linear decomposition of the depth of coverage matrix into effects due to GC content, exon capture and amplification and other latent systemic factors. The normalization model produces an estimated ‘control coverage’ for each exon and each sample, which is the coverage we expect to see if there is no CNV. Next, the observed coverage for each exon and each sample is compared to the corresponding estimated control coverage in a Poisson likelihood-based segmentation algorithm, which returns a segmentation of the genome into regions of homogeneous copy number. A direct estimate of the relative copy number, in terms of fold change from the expected control value, can be used for genotyping. Heatmaps of raw depth of coverage in an example region, its corresponding normalized coverage and its segmentation results are shown in Figure 1. CODEX is freely available as a Bioconductor R package at http://www.bioconductor.org/packages/devel/bioc/html/CODEX.html.

### Sample selection and target filtering

To have as much sample- and exon-wise homogeneity as possible and to make sure that our normalization algorithm converges without being deviated by extreme values, we adopt a sample selection and target filtering strategy before applying our proposed normalization method to the read depth data. Specifically, for reducing artifacts, we recommend that all of the samples be sequenced by the same platform. We further filter out exons that: (i) have extremely low coverage (median read depth across all samples less than 20, which mostly reflect capture failure); (ii) are extremely short (less than 20 base pairs); (iii) are hard to map (mappability less than 0.9, Supplementary Figure S1); (iv) have extreme GC content (less than 20% or greater than 80%). These default thresholds for quality control (QC) are recommended but are also user-tuneable and thus can be adapted to different sequencing protocols. We show in Table 1 that with the above QC thresholds, 9.74% of exon targets are excluded in the data. Details on computation of GC content, mappability and depth of coverage are provided in Supplementary Material.

**Table 1.** CNV call sets information on the 1000 Genomes Project WES data set

Chr	Number of targets (before/after QC)	CODEX		XHMM		CoNIFER		EXCAVATOR CNVs
		*K*	CNVs	PCs	CNVs	PCs	CNVs	
1	15 426/14 101	3	361 (301-60)	4	129 (56-73)		36 (13-23)	263 (236-27)
2	9640/8956	3	54 (14-40)	4	51 (16-35)		6 (0-6)	15 (0-15)
3	8267/7775	3	13 (0-13)	4	8 (0-8)		4 (0-4)	5 (0-5)
4	5519/5157	4	27 (16-11)	4	20 (7-13)		16 (6-10)	91 (86-5)
5	6403/5950	3	163 (143-20)	4	39 (23-16)		5 (0-5)	79 (72-7)
6	6997/6569	3	115 (95-20)	4	34 (11-23)		15 (6-9)	62 (58-4)
7	6210/5546	3	164 (118-46)	4	62 (24-38)		6 (0-6)	121 (108-13)
8	4477/4118	3	51 (42-9)	4	12 (0-12)		2 (0-2)	41 (39-2)
9	5777/5136	3	27 (6-21)	4	24 (0-24)		7 (0-7)	66 (42-24)
10	6354/5759	3	28 (6-22)	4	27 (9-18)		6 (0-6)	55 (50-5)
11	7778/6979	3	77 (54-23)	4	26 (0-26)		7 (0-7)	73 (45-28)
12	7817/7261	3	35 (6-29)	4	32 (2-30)	4	9 (0-9)	25 (12-13)
13	2536/2362	3	14 (0-14)	4	7 (0-7)		0 (0-0)	3 (0-3)
14	4482/4127	3	37 (29-8)	4	16 (0-16)		8 (7-1)	56 (46-10)
15	4635/4150	3	93 (66-27)	4	40 (18-22)		5 (0-5)	73 (65-8)
16	5596/4744	4	154 (124-30)	5	86 (57-29)		9 (0-9)	112 (78-34)
17	8283/7386	3	91 (58-33)	4	49 (23-26)		10 (0-10)	124 (110-14)
18	2021/1888	3	4 (0-4)	4	5 (0-5)		1 (0-1)	3 (0-3)
19	7438/5982	4	168 (117-51)	5	135 (103-32)		15 (0-15)	197 (131-66)
20	3966/3497	3	11 (7-4)	4	9 (0-9)		1 (0-1)	18 (7-11)
21	1499/1314	4	4 (0-4)	4	29 (26-3)		0 (0-0)	61 (58-3)
22	2957/2493	4	79 (62-17)	5	55 (38-17)		12 (0-12)	124 (107-17)
X	5436/4787	3	36 (15-21)	4	60 (41-19)		9 (0-9)	248^a^ (248^a^-0)
Y	281/146	3	0 (0-0)	3	0 (0-0)		0 (0-0)	144^a^ (144^a^-0)
Sum	139 795/126 183	-	1806 (1279-527)	-	955 (454-501)	-	189 (32-157)	1667 (1350-317)

Number of exon targets before and after QC procedure is shown. CNVs detected by CODEX, XHMM, CoNIFER and EXCAVATOR are shown and are further categorized into common and rare ones (common–rare in parentheses). Number of latent factors (*K*) and PCs are shown for latent factor models: default values from CODEX and XHMM are adopted; number of PCs for CoNIFER is chosen at 4 so that it is conservative by the scree plot and is comparable to XHMM.

^a^Excluded due to mishandling of sex chromosomes by EXCAVATOR.

### Read depth normalization

Due to the extremely high level of systemic bias in WES data, normalization is crucial in WES CNV calling. CODEX's multi-sample normalization model takes as input the WES depth of coverage, exon-wise GC content and sample-wise total number of reads. Specifically, we denote *Y* as the coverage matrix with row *i* (1 ≤ *i* ≤ *n*) corresponding to the *i*th exon and column *j* (1 ≤ *j* ≤ *m*) to the *j*th sample, *GC_i_* as the GC content for exon *i* and *N_j_* as the total number of mapped reads for sample *j*. The ‘null’ model, which reflects the expected coverage when there is no CNVs, is

\begin{equation*} \begin{array}{*{20}l} {Y_{ij} \sim{\rm Poisson}(\lambda {}_{ij})} \\ {\lambda _{ij} = N_j f_j (GC_i )\beta _i \exp \left( {\sum\limits_{k = 1}^K {g_{ik} h_{jk} } } \right),} \\ \end{array} \end{equation*}

where *f_j_*(*GC_i_*) is the bias due to GC content for exon *i* sample *j*; *β_i_* reflects the exon-specific bias due to length and capture and amplification efficiency of exon *i* and *g_ik_h_jk_* (1 ≤ *k* ≤ *K*) are the *k*th latent Poisson factors for exon *i* and sample *j*. The goal of fitting the null model to the data is to estimate the various sources of biases, which can then be used for normalization.

We adopt a robust iterative maximum-likelihood algorithm for estimating the parameters of the null model. Briefly, in each iteration, we estimate *f*(*GC*) by fitting a smoothing spline of ${Y / {N\beta \exp (g \times h^{\rm T} )}}$ against the GC content, using the built-in function *smooth.spline* in R. *β* takes the value of the median of each row in ${Y / {Nf(GC)\exp (g \times h^{\rm T} )}}.$ The latent variables $g_{ik} h_{jk}$ (1 ≤ *k* ≤ *K*) are estimated in the following steps: (i) take known *h* as covariates, fit *n* Poisson log-linear regressions with each row of *Y* as the response and corresponding row of $\log \left( {Nf(GC)\beta } \right)$ as the fixed offset; (ii) take known *g* as covariates, fit *m* Poisson log-linear regressions with each column of *Y* as the response and corresponding column of $\log \left( {Nf(GC)\beta } \right)$ as the fixed offset; (iii) apply SVD to the row-centered matrix *g* × *h*^T^ to obtain the *K* right singular vectors to update *h*. The third step ensures the uniqueness and orthogonality of the updated components, which forces the identifiability of $g_{ik} h_{jk}$ (1 ≤ *k* ≤ *K*) (36). We fit the Poisson log-linear models with the built-in function *glm* in R. See below for details of the maximum-likelihood algorithm. Procedures for determining *K*, the number of latent Poisson factors, is discussed later.

Initialization

$\beta ^{old} = 1^n,\;\; g = 0^{n \times K},\;\; h = 0^{m \times K}.$

*Iteration*

- For each sample *j*, fit a smoothing spline of $\frac{{Y_{j} }}{{N_j \beta ^{{\rm old}} \left( {{\rm exp}(g \times h^{\rm T} )} \right)_{j} }}\sim GC$ to get $f_j (GC).$
- For each exon *i*, update *β_i_* as $\beta _i^{{\rm new}} = {\rm median}\left( {\left[ {\frac{Y}{{Nf(GC)\exp (g \times h^{\rm T} )}}} \right]_{i} } \right).$
- Denote $Z = Nf(GC)\beta ^{{\rm new}} .$ Apply SVD to row-centered $\log ({Y / Z})$ to obtain the *K* right singular vectors and use as $h^{{\rm old}} .$
  - Fit *n* Poisson log-linear regressions with *Y_i_*_·_ as response, *h^dd^* as covariates, $\log (Z_{i} )$ as fixed offset to obtain updated estimates as *g*.
  - Fit *m* Poisson log-linear regressions with $Y_{j}$ as response, *g* as covariates, $\log (Z_{j} )$ as fixed offset to obtain updated estimates as *h*^new^.
  - Center each row of $g \times (h^{{\rm new}} )^{\rm T}$ and apply SVD to the row-centered matrix to obtain the *K* right singular vectors to update *h*^new^.
  - Repeat steps a to c with $h^{{\rm old}} = h^{{\rm new}}$ until convergence to obtain *h* and *g*.
- Repeat steps i to iii with $\beta ^{{\rm old}} = \beta ^{{\rm new}}$ until convergence.

After the normalization procedure, we obtain $\widehat\lambda = N\widehat\beta \widehat{f}(GC)\exp \left( {\widehat{g} \times \widehat h^{\rm T} } \right),$ which is the expected ‘control coverage’ in the event where there is no CNV. As described later, the observed coverage *Y* will be compared to the corresponding estimated control coverage $\widehat\lambda$ to test for the presence of CNVs.

For CNV detection under case–control settings (e.g. tumor with normal) involving recurrent large chromosomal aberrations, CODEX estimates the exon-wise Poisson latent factor $\{ h_{jk} \}$ using only the read depths in the control cohort, and then computes the {*g_ik_*} terms for the case samples by regression. This leads to higher sensitivity for detecting variants that are present only in the case samples. CODEX also includes two modes—‘integer’ mode that returns copy numbers as integers for germline CNV detection and ‘fraction’ mode that returns fractional copy numbers for CNV detection of samples with heterogeneous genetic compositions.

### Poisson latent factors and choice of *K*

Some sources of bias in WES can be directly measured (GC content, mappability and exon size). However, there are other unmeasurable sample- and target-specific biases that are amplified during the library preparation and sequencing experiment. The latent Poisson factors $\{ g_{ik} \}$ and $\{ h_{jk} \}$ are designed to capture and decompose these unobserved systemic bias in a log-additive manner. Such latent factor models have been shown to be effective in the analysis of microarray data (37–40), and have also recently been applied to NGS data. Both CoNIFER (26) and XHMM (28) use latent factor models to remove systemic bias, but their models assume continuous measurements with Gaussian noise structure, while CODEX is based on a Poisson log-linear model, which is more suitable for modeling the discrete counts in WES data, especially when there is high variance in depth of coverage between exons. The latent factor terms in the normalization model resemble those used in Lee *et al*. (36) for microRNA profiling. In particular, the identifiability constraints in Lee *et al*. also apply to our case, and our iterative maximum-likelihood estimation procedure ensures identifiability.

A common downfall of latent factor models is that true CNV signals may correlate with and influence the top *K* latent factors. Thus, the number of latent factors, *K*, is a crucial parameter. If *K* is chosen to be too large, some *bona fide* CNV signals, especially those for common CNVs, will be dampened during normalization. On the other hand, if *K* is too small, residual artifacts will remain and inflate the type I error rate. CoNIFER (26) adopts a common practice for choosing the number of factors in latent variable models, which is to draw the screen plot with the number of components on the *X*-axis and the corresponding contributed variance on the *Y*-axis. If there is an ‘elbow’ in the scree plot, then *K* is chosen at the position of the elbow. However, in most cases there is no detectable elbow, which is why many existing methods arbitrarily set the value of *K*. XHMM removes components with variance 0.7/*m* or higher, where *m* is the number of components (samples) and 0.7 is a user-tuneable parameter arbitrarily set as default (28).

We apply two additional statistical procedures of choosing this crucial model tuning parameter: Akaike information criterion (AIC) and Bayes information criterion (BIC)

\begin{equation*} \begin{array}{*{20}l} {{\rm AIC} = 2\ln (L) - 2k} \\ {{\rm BIC} = 2\ln (L) - k\ln (n),} \\ \end{array} \end{equation*}

where *L* is the likelihood for the estimated model, *k* is the number of parameters in the model and *n* is the number of data points. Both criteria reward goodness of fit with a penalty term that is an increasing function of the number of parameters in the model. AIC penalizes the number of parameters less strongly than does BIC, and thus the model chosen by AIC removes more latent factors than that chosen by BIC. CODEX reports all three statistical metrics (AIC, BIC, percentage of variance explained) and uses BIC as the default method to determine the number of *K*. Since false positives can be screened out through a closer examination of the post-segmentation data, whereas CNV signals removed in the normalization step cannot be recovered, CODEX opts for a more conservative normalization that, when in doubt, uses a smaller value of *K*.

### CNV detection and copy number estimation

Proper normalization sets the stage for accurate segmentation and CNV calling. For germline CNV detection in normal samples, many CNVs are short and extend over only one or two exons. In this case, simple gene- or exon-level thresholding is sufficient.

For longer CNVs and for copy number estimation in tumors where the events are expected to be large and exhibit nested structure, we propose a Poisson likelihood-based recursive segmentation algorithm. Let $y_s ,...,y_t$ and $\lambda _s ,...,\lambda _t$ be the raw and estimated control coverage of the window spanning exon *s* to exon *t*. The values $\lambda _s ,...,\lambda _t$ are estimated by the normalization procedure described in the previous section, but suppressing the sample indicator *j* since we segment each sample separately. A joint cross-sample segmentation, as proposed in Zhang *et al*. (41), can also be applied and may yield more accurate results for detection of germline CNVs. Let $y_{s:t} = \sum\limits_{i = s}^t {y_i }$ and $\lambda _{s:t} = \sum\limits_{i = s}^t {\lambda _i } .$ The scan statistic we use is $\max _{s,t} U(s,t),$ where

\begin{equation*} \begin{array}{*{20}l} {U(s,t) = \mathop {\sup }\limits_\mu \left( {\log \left( {\frac{{\mu ^{y_{s:t} } \exp ( - \mu )}}{{\lambda _{s:t} ^{y_{s:t} } \exp ( - \lambda _{s:t} )}}} \right)} \right) = } \\ {y_{s:t} \log \left( {\frac{{y_{s:t} }}{{\lambda _{s:t} }}} \right) - (y_{s:t} - \lambda _{s:t} )} \\ \end{array} \end{equation*}

The above is the generalized log-likelihood ratio of the alternative model, $y_{s:t} \sim {\rm Poisson}(\mu )$ with *μ* arbitrary, versus the null model, $y_{s:t} \sim {\rm Poisson}(\lambda _{s:t} ).$ The copy number estimate for the window is given by $2{{y_{s:t} } / {\lambda _{s:t} .}}$

Given the scan statistic, CODEX performs a circular binary segmentation procedure (42) using *U*(*s*,*t*). We further use a modified BIC (mBIC) to determine the number of change points *P* in our model (43),

\begin{equation*} \begin{array}{*{20}l} {{\rm mBIC}(P) = } \\ {\log \left( {\frac{{L_\tau }}{{L_0 }}} \right) - \frac{1}{2}\sum\limits_{\rho = 0}^P {\log (\widehat\tau _{\rho + 1} - \widehat\tau _\rho ) + (\frac{1}{2} - P)\log (n),} } \\ \end{array} \end{equation*}

where the first term is the generalized log-likelihood ratio for the model with *P* change points versus the null model with no change points; $\tau _\rho$ $(1 \le \rho \le P)$ is the $\rho {\rm th}$ change point, $1 = \tau _0 < \tau _1 < ... < \tau _P < \tau _{P + 1} = n;$ *n* is the number of exons. We report the segmentation with $\widehat{P} = {\rm argmax}_P {\rm mBIC}(P).$ Compared with algorithms based on HMM, such as XHMM and EXCAVATOR, CODEX does not require the user to pre-specify unknown parameters, such as expected distance between exons, exon-wise CNV rate and average number of exons in a CNV. These quantities are often hard to set *a priori* without a large relevant training data set, and in many cases have to be chosen arbitrarily. Post-segmentation, CODEX outputs an estimate of the relative copy number in terms of fold change from the expected control coverage, rather than a binary categorization of deletion and duplication as in CoNIFER (26) and XHMM (28).

### Samples and data sets

To examine the accuracy of CODEX and to illustrate its application, we use a publicly available WES data set from the 1000 Genomes Project Phase 1 release (29) containing 90 healthy individuals. Forty-six samples are sequenced at the Washington University Genome Sequencing Center (captured by HSGC VCRome) and 44 at the Baylor College of Medicine (captured by SureSelect All Exon V2). All samples have Omni and Axiom genotypes and have more than 70% of exome targets covered to 20× or more. Sex is well balanced (44 males and 46 females) and population (40 Utah residents with northern and western European ancestry (CEU), 24 Japanese people from Tokyo (JPT) and 26 Yoruba people from Ibadan (YRI)) adds a potential source of latent variation. More details on the HapMap samples are provided in Supplementary Table S1.

We also analyze a WES data set consisting of 222 paired tumor/normal (blood leukocyte) samples of individuals older than 18 months of age at diagnosis with stage-4 neuroblastoma from the TARGET Project (20). WES of native and whole genome amplified DNA of ∼33 Mb regions yields a 124× average coverage, with 87% of bases suitable for mutation detection (20).

## RESULTS

### Calling germline variations from HapMap samples

#### Effectiveness of normalization procedure

**Figure 3.**
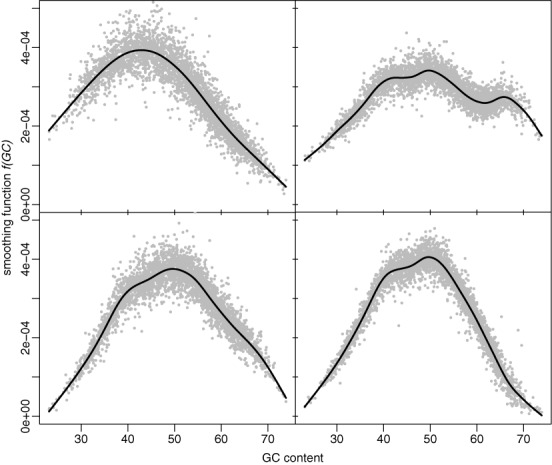
Predicted values of $f(GC)$ for four samples from the 1000 Genomes Project data set. Most patterns agree with previous observations that read depth has a unimodal relationship with GC content. However, dual modality is also observed. Furthermore, the function changes in shape and not just by a scaling factor.

We first examine the effectiveness of CODEX's proposed normalization model on the 1000 Genomes Project WES data set (29). Previous studies have shown that read depth has a unimodal relationship with GC content—regions with high or low GC content tend to have decreased read depth (44). In our smoothed estimates of $f_j (GC),$ we find that most but not all samples have a unimodal shape for this function. We show the predicted values of $f_j (GC)$ for four typical samples in Figure 3. Interestingly, we found that some samples have estimates with multiple peaks in $f_j (GC),$ which suggests that a parametric functional form assuming unimodality may be too simplistic. Comparing across samples, we see that the function $f_j (GC)$ changes in shape and not just by a scaling factor. Therefore, the GC content bias is not linear across samples and thus cannot be fully captured by linear latent factor models. This motivates the separate non-parametric term in our model for GC bias.

We further compare the normalization result of CODEX against that of SVD-based method using array-based CNV calls from the International HapMap Consortium (30) on the same samples we analyze. For different categories of CNV events, namely, homozygous deletions, heterozygous deletions and duplications, we use direct thresholding of $\log ({Y / {\widehat\lambda }})$ to draw receiver operating characteristic (ROC) curves of our model, where $\widehat\lambda$ is the estimated control coverage from CODEX's normalization procedure. The ROC curves for SVD-based normalization are drawn by thresholding on the residuals obtained by subtracting the first *K* principal components (PCs) from the original read depth *Y*. A separate power analysis is done for each of the following category of events: common homozygous deletion, common heterozygous deletion, common duplication, rare heterozygous deletion and rare duplication (Supplementary Figure S2). There are no rare homozygous deletions as all of the rare deletions from the HapMap CNV call set are present in only heterozygous form. We see that CODEX's normalization procedure leads to a better signal-to-noise ratio for both common and rare CNVs, and for both deletions and duplications (Supplementary Figure S2).

#### Accuracy of CNV calling

We next compare the accuracy of CODEX to existing approaches that are designed for population-based CNV calling. These programs include CoNIFER (26), XHMM (28) and EXCAVATOR (25) in its ‘pooling’ mode, for which we added four additional samples as controls (Supplementary Table S1).

**Figure 4.**
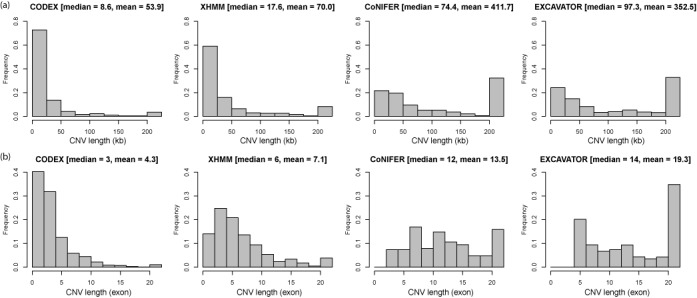
Lengths of CNV calls by CODEX, XHMM, CoNIFER and EXCAVATOR. Genomics lengths of CNVs (a) and number of exons in CNV regions (b) are compared across four different methods. CODEX and XHMM detects more short CNVs, whereas CoNIFER and EXCAVATOR return significant proportion of CNVs with lengths greater than 200 kb/20 exons.

The number of calls made by each program on each chromosome sample, broken down into common and rare calls, is shown in Table 1. Globally, CODEX detects twice as many CNV events as XHMM does and nearly 10 times as many as CoNIFER does, while EXCAVATOR and CODEX have comparable number of calls. CoNIFER detects the fewest CNVs in total, which agrees with comparisons against EXCAVATOR made in Magi *et al*. (25). Since CoNIFER does not automatically choose the number of PCs, we fix the number of PCs filtered out by CoNIFER at 4, agreeing with the selection made by XHMM so as to make the two SVD-based programs comparable. The choice of 4 PCs in normalization should not account for the low number of calls made by CoNIFER, since through the scree plot output by CoNIFER, we find the curve of relative contributed variance to be still significantly decreasing at 4, indicating that the choice of 4 is conservative (Supplementary Figure S3). A large proportion of XHMM and CoNIFER calls are rare (<5%) variants—52.46% (501/955) and 83.07% (157/189), respectively. Despite the bias in sensitivity of HMM and CoNIFER toward rare variants, CODEX detects even more rare CNVs in total as well as proportionately more common ones. Notably, the number of latent factors *K* selected by CODEX is for most chromosomes one less than the number of PCs excluded by XHMM across the genome. Furthermore, CODEX and XHMM tends to detect shorter CNVs compared to CoNIFER and EXCAVATOR in units of both kb (Figure 4a) and exon (Figure 4b). Detailed CNV call sets by the four methods are provided in Supplementary Table S2.

**Figure 5.**
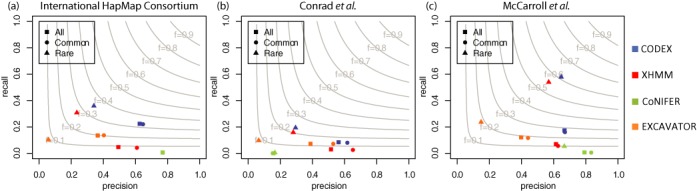
Assessment of CNV calls on the 1000 Genomes Project by array-based methods. CNV calls by CODEX, XHMM, CoNIFER and EXCAVATOR are validated against genotyping calls from International HapMap Consortium (a), Conrad *et al*. (b) and McCarroll *et al*. (c). CODEX returns well-balanced precision and recall rates with highest *F*-measures (gray contours shown harmonic means of precision and recall rates) among all methods for detection of common, rare and all CNVs.

We assess the CNV calls made by the four methods by comparing to calls reported by the International HapMap Consortium (30), McCarroll *et al*. (31) and Conrad *et al*. (32) in the same 90 HapMap samples. The International HapMap 3 Consortium produced a clean CNV call set by merging and utilizing probe-level intensity from both Affymetrix and Illumina arrays, containing 856 copy number polymorphisms (CNPs) with a 99.0% mean call rate and 0.3% Mendelian inconsistency (30). Separately, McCarroll *et al*. developed a map consisting of 1320 CNVs at 2-kb breakpoint resolution by joint analysis of Affymetrix SNP array, array CGH (45) and fosmid end-sequence-pair data (31,46). The third source of validation we use is the call set from Conrad *et al*., who used Nimblegen tiling oligonucleotide arrays to generate a map of 11 700 CNVs greater than 443 base pairs, of which 8599 have been validated independently (32). The genotyped CNPs from these three cohort studies that overlap with exon regions (73, 123 and 377 in total, respectively) are used as ‘validation set’ to assess sensitivity and specificity of the four methods compared in Table 1 (details provided in Supplementary Table S3). Figure 5 shows the precision and recall rates (precision is the proportion of calls made by the program that overlap with validation set, and recall is the proportion of the CNVs in validation set that are called.) The different programs vary considerably in precision and recall rate. CODEX has the highest *F*-measure (harmonic mean of precision and recall) for both common and rare CNVs (Figure 5). XHMM performs well in detecting rare variants but is insensitive to common ones (Figure 5). CoNIFER has the highest precision when comparing against calls from the International HapMap Consortium (Figure 5a) and McCarroll *et al*. (Figure 5c) but gives poor results against Conrad *et al*. (Figure 5b). Furthermore, the high precision of CoNIFER come with significant sacrifice on recall (Figure 5). See Supplementary Table S4 for detailed comparison results based on the three SNP array metrics.

#### Sensitivity assessment with spike-in study

We next conduct an *in silico* spike-in study to assess the sensitivity of the different methods at varying population frequencies. Starting with the WES data from chromosome 20 of the *m* = 90 HapMap samples analyzed in the previous section, we spike CNV signals in to copy-number-neutral regions. We define a region to be copy-number-neutral if it does not overlap with CNV calls made by CODEX, XHMM, EXCAVATOR and CoNIFER nor with previously reported CNV regions by DGV (http://dgv.tcag.ca/dgv/app/) and dbVar (http://www.ncbi.nlm.nih.gov/dbvar/). Of the 3966 exon targets on chromosome 20, 1035 pass this criterion for copy-number-neutral. We considered only heterozygous deletions of two different lengths (5 and 10 exons) and varying population frequencies ($p \in \{ 5\% ,10\% , \ldots ,95\% \}$). We focus on heterozygous deletions because (i) homozygous deletions are easily detectable by all methods; (ii) as is shown in Supplementary Figure S4, heterozygous deletions with frequency *p* in the population have exactly the same detection accuracy as duplications with frequency $1 - p.$ Specifically, for deletions with population frequencies greater than 50%, copy-number-neutral states are reported as duplications, whereas deletions are reported as normal events, since all copy number events are defined in reference to a population average. Events are centered at every hundredth exon and *m* × *p* samples are randomly chosen to be carriers. To generate CNV signals for heterozygous deletions, we reduce the raw depth of coverage for exons spanned by the CNV from *y* to $\frac{c}{2} \times y$, where *c* is sampled from a normal distribution with mean 1 and standard deviation 0.1.

**Figure 6.**
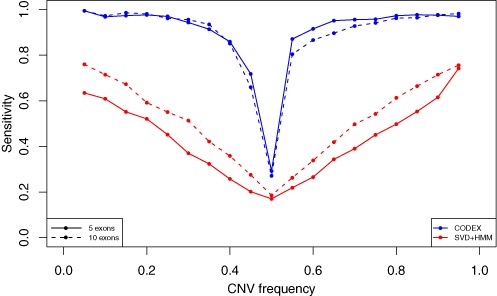
Power analysis of CODEX and SVD-based method on simulation data set. Sensitivities are obtained by averaging results from 10 simulations. Both methods suffer from ‘common’ CNV events (CNVs with frequencies around 50%). When CNV frequency exceeds 50%, deletions and copy-neutral states are detected as copy-neutral states and duplications instead, which recovers the sensitivities. CODEX performs better compared to SVD-based methods with higher power. Longer CNVs are generally easier to be detected.

We apply CODEX to these spike-in data sets and compare it to SVD-based normalization followed by HMM-based segmentation. For the latter, we remove the first *K* PCs from the read depth matrix and transform the residuals to *z*-scores for each sample separately. The *z*-scores are then segmented by a HMM whose parameters are set as the default values in XHMM. The specificity of both approaches is controlled to be higher than 99%. The sensitivities for short CNV (5 exons) and long CNV (10 exons) at different population frequency levels are shown in Figure 6. We see that both approaches attain high sensitivity for rare CNVs, and both have decreased sensitivity for common CNV events. The sensitivity of CODEX is higher than that of the existing approach for both rare and common variants (Figure 6, Supplementary Figure S5). For CNV events with frequencies around 50%, both methods have the lowest power due to the fact that the CNV signals are falsely filtered out by a sample-wise latent factor (Figure 6, Supplementary Figures S5 and S6). Also, shorter CNV events are more often missed by the SVD approach, whereas CODEX has comparable sensitivity for short and long variants at this scale (Figure 6, Supplementary Figure S5). We also examine the effect of different choice of QC procedures and Supplementary Figure S5 shows that detection power indeed suffers from not removing outstanding outliers.

To gain a better understanding of what the latent factors in CODEX and SVD-based methods are capturing, we show in Supplementary Figure S6 the correlation of the latent factors to measurable quantities. The exon-wise latent factors in both models and the estimated value of *β* in CODEX are compared to GC content, mean exon coverage and true copy number. The sample-wise latent factors in both models are compared to center, batch, population and total coverage (*N*). Based on these correlations, we make the following observations: First, mean exon coverage (represented by the pseudo-reference sample $\left \{ \left ( \prod \nolimits _{v=1}^{m}\; Y_{iv} \right )^{1/m}:1 \leq i \leq n \right \}$) is captured by *β* in (correlation coefficient 0.99) in CODEX and the first exon-wise PC in SVD (correlation coefficient −0.98). Exon length and capture and amplification efficiency are confounded in this exon-specific bias and there is no way, nor any need, to estimate these individual quantities separately. Second, GC content is correlated with the third exon-wise PC in SVD (correlation coefficient −0.75). CODEX specifically models the GC content bias for each sample by the term $\left\{ {f_j (GC):1 \le j \le m} \right\},$ and as we show later, the bias cannot be fully captured by a linear PC. Third, a CNV that is more frequent in the population has higher absolute correlation between copy number state and the exon-wise latent factors in both CODEX (−0.22) and SVD (0.57). This is why sensitivity is lower for common CNVs. Finally, other known sources of bias, such as sequencing center and batch, are captured by sample-wise latent factors in both CODEX (correlation coefficient −1 and 0.74) and SVD (correlation coefficient 0.97 and −0.71). In this data set, population does not seem to be captured by any of the top latent factors.

### Analysis of WES of neuroblastoma

We also apply CODEX to the WES data of 222 neuroblastoma patients from the TARGET Project (20). Our discussion here focuses on the well-characterized *ATRX* gene region (20,33,34). The TARGET Project reported recurrent focal deletions with a complex nested structure spanning the *ATRX* gene. Since there are matched normal samples for this study that have also been sequenced by the same technology, the TARGET calls were made by comparing each tumor sample to its matched normal. This allows us to compare the effectiveness of CODEX's normalization model to that of taking a log ratio to the matched normal coverage. Also, focusing on this well-characterized region allows us to demonstrate in accuracy of CODEX for handling recurrent complex nested events.

**Figure 7.**
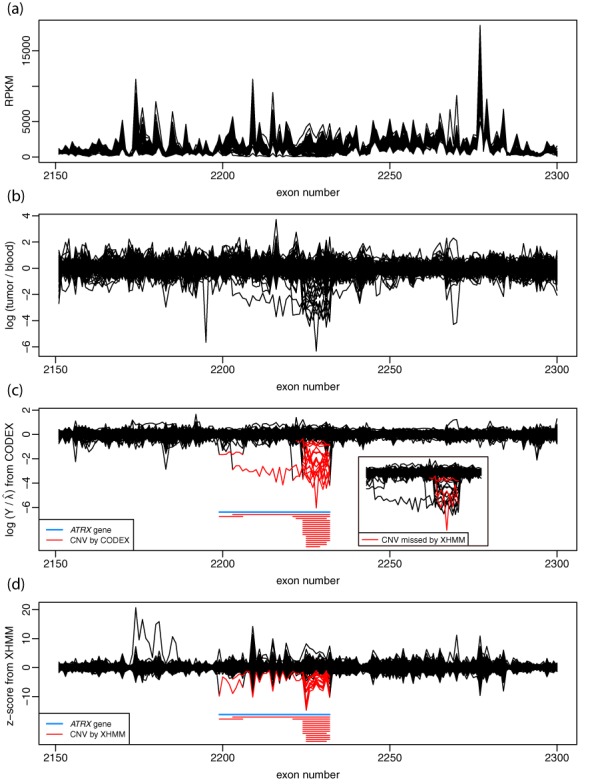
Detection of rare somatic deletions within *ATRX* by WES of 222 neuroblastoma-matched tumor/blood samples. Location of *ATRX* is shown as blue bars in c and d. (a) RPKM computed from the tumor samples. There is no clear visual indication of presence of somatic CNVs from these raw quantities. (b) Log_2_-ratio of tumor versus blood read depth. Initial analysis by the TARGET Project did careful inspection of these values and discovered 16 samples with focal deletions. (c) Log_2_-ratio of the original tumor read depth *Y* versus the estimated control coverage $\widehat\lambda$ (model fitted on tumor data set only) by CODEX. Poisson likelihood-based segmentation algorithm by CODEX discovers 18 samples (red bars) with somatic deletions that exhibit a nested structure across samples. The 4 samples that are called by CODEX but not by XHMM are colored in red in the embedded window. (d) XHMM's direct output: *z*-scores normalized by PC analysis. The HMM calling algorithm by XHMM detects 14 samples (red bars) with somatic deletions.

The RPKM (reads per kilo bases per million reads) for each exon and each sample are plotted in Figure 7a. The RPKM profiles are very noisy and do not show any clear decrease in this region in any of the samples, highlighting the need for normalization. For comparison, we also show the TARGET Project's initial analysis, which reported 16 multi-exon deletions within *ATRX* by comparing tumor to matched normal samples (20). Specifically, we repeat their analysis by thresholding the log_2_-ratio of RPKM in tumor to RPKM in normal samples, illustrated in Figure 7b. Figure 7c shows the normalized intensities given by CODEX, which detects 18 samples with somatic focal deletions (plots for each individual sample are given in Supplementary Figure S7). We also apply XHMM to the tumor data set and detect 14 samples with focal deletions (Figure 7d).

Of the 18 samples with somatic deletions detected by CODEX, three are also called by the TARGET Project but missed by XHMM; one is detected by XHMM and CODEX with exactly the same breakpoints but is missed by the Target Project; one is uniquely called by CODEX (Supplementary Table S5a). The sample uniquely called by CODEX is a small deletion that overlaps significantly with deletions called in other samples. Detailed CNV calling and genotyping results by each method are in Supplementary Table S5b–d and the genome-wide blood and tumor CNV events discovered by CODEX are summarized in Supplementary Table S6. The comprehensive analysis results will be published separately.

It is clear by visual comparison of Figure 7c to b and d that the read depth normalization method within CODEX gives better signal-to-noise ratio than the SVD-based normalization method in XHMM (note the difference in range of the *y*-axes) and also better than the commonly prescribed method of normalizing to matched normal controls. This illustrates that by borrowing information across a large cohort, the estimated control coverage of $\widehat\lambda$ from our normalization model is more effective in capturing the biases in WES than the matched normal. Whereas the matched normal sample is important to distinguish between germline and somatic variants, CODEX's normalization procedure can be used in case of unavailability of blood samples or contamination of blood samples from circulating tumor cells. When matched normal is available, somatic status can be determined by comparing CODEX calls in tumor to those in normal. This example also shows that CODEX's segmentation algorithm performs well in detecting multi-exon CNVs with a nested structure, and that it successfully detected a rare CNVs (18/222 = 8.11%) in a clinical setting.

## DISCUSSION

Here we propose CODEX, a normalization and CNV detection method for WES data. CODEX includes a normalization model with non-parametric functional terms for GC content and Poisson latent factors for biases that are not directly quantifiable. We show that both parts of the normalization model are necessary for WES data. CODEX segments the genome using a Poisson likelihood model based on the control coverage $\widehat\lambda$ estimated during the normalization step. CODEX can be applied to both normal and tumor genome analysis.

We show through several data sets that CODEX's multi-sample normalization procedure offers higher sensitivity and specificity for detection and genotyping of both common and rare CNVs. The distinguishing features of CODEX compared to existing methods are: (i) CODEX does not require matched normal samples as controls for normalization; (ii) The Poisson log-linear model fits better with the WES count data than SVD approaches; (iii) Dependence on GC content is modeled by a flexible non-parametric function in CODEX allowing it to capture non-linear biases; (iv) CODEX implements the BIC criterion for choosing the number of latent variables, which gives a conservative normalization on simulated and real data sets; (v) Compared to HMM-based segmentation procedures, the segmentation procedure in CODEX is completely off-the-shelf and does not require large relevant training set; (vi) CODEX estimates relative copy number, which can be converted to genotypes by thresholding, rather than broad categorizations (deletion, duplication and copy number neutral states).

We carry out simulation studies by spiking in CNV signals to WES read depth data from copy-number-neutral regions. We show that CODEX has higher power compared to SVD-based method followed by HMM, although both methods suffer from common CNV events. We also investigate the nature of the exon- and sample-wise terms and Poisson factors in CODEX, PCs extracted by SVD and other directly known biases and artifacts. We show that PCs from SVD obtained by unsupervised learning are correlated by the terms specifically modeled and quantified by CODEX and that the GC content correlates with one PC from SVD with correlation coefficient −0.75, which, again, is specifically modeled by CODEX. Developing a robust method that can detect common CNVs from background noise with high sensitivities may be a future direction to get focused on.

We compare CODEX's performance against direct calling results from other existing methods on the 1000 Genomes Project WES data set and show that CODEX is more accurate by comparing CNV calls by WES against three gold standard SNP array CNV call sets. Since CoNIFER and EXCAVATOR detect a significant proportion of CNVs with lengths greater than 200 kb, whereas CODEX and XHMM return much shorter CNVs (Figure 4), we do not exclude any CNV calls by SNP arrays so as to get more ‘reliable’ gold standards as does Fromer *et al*. (28), despite the fact that array-based methods, when compared to NGS, do not have as good resolutions. This might explain why the overall sensitivity/recall rates are no larger than 0.6 for all methods (Figure 5, Supplementary Table S4). Another possible explanation lie in that due to the discrete nature of WES data, read depth is used as the only inference to detect CNVs, which has only exon-level resolution and thus lower power in detecting short CNVs compared to split-read and paired-end-mapping methods developed for WGS. Despite the limitations, WES has been used and is still being used as a preferred method of choice for large-scale studies.

With a clinically relevant example on detecting rare somatic CNVs within *ATRX* associated with neuroblastoma, CODEX is shown to be applicable to a wide range of study designs for CNV detection using WES data. Specifically, we show that CODEX does not require matched normal controls for normalization and is able to detect previously reported CNVs within tumor samples more accurately compared to SVD-based method. Matched blood samples, when available, can be used to distinguish somatic CNVs from germline ones. However, under most circumstances, the normal samples are often unavailable, incomplete or unmatched, which drives the need for normalization using cases only. The genome-wide CNV results based on this data set are available and will be compared against other metrics (matched microarrays, whole-genome sequencing, RNA-sequencing, etc.) and validated on bench. The comprehensive analysis results will be published elsewhere.

## SUPPLEMENTARY DATA

Supplementary Data are available at NAR Online.

SUPPLEMENTARY DATA
