# Supplementary material for: CODEX: a normalization and copy number variation detection method for whole exome sequencing
Source: Nucleic Acids Res. 2015 Jan 23;43(6):e39. doi: 10.1093/nar/gku1363 (PMC4381046; doi:10.1093/nar/gku1363)
Supplement: SUPPLEMENTARY DATA [file supp_43_6_e39__index.html]

CODEX: a normalization and copy number variation detection method for whole exome sequencing — SUPPLEMENTARY DATA 

# CODEX: a normalization and copy number variation detection method for whole exome sequencing

## SUPPLEMENTARY DATA

**Files in this Data Supplement:**

- SUPPLEMENTARY DATA
- SUPPLEMENTARY DATA
- SUPPLEMENTARY DATA
