# Supplementary material for: CODEX: a normalization and copy number variation detection method for whole exome sequencing
Source: Nucleic Acids Res. 2015 Jan 23;43(6):e39. doi: 10.1093/nar/gku1363 (PMC4381046; doi:10.1093/nar/gku1363)
Supplement: SUPPLEMENTARY DATA [file supp_gku1363_nar-01605-met-n-2014-File010.docx]

**Supplementary Material**

**CODEX: a normalization and copy number variation detection method for whole exome sequencing.**

Yuchao Jiang, Derek A Oldridge, Sharon J Diskin, Nancy R Zhang

**Supplementary methods**

***Depth of coverage, GC content, and mappability***

Depth of coverage for each exon is computed as the number of reads (with mapping quality greater than a user-defined threshold) that overlap with the exon. To calculate the exonic mappability, we first construct consecutive reads that are one base pair (bp) apart along the exon. The length of the reads is set to be the same as that from the sequencing technology and the sequences are taken from the hg19 reference. We then find possible positions across the genome that the reads can map to allowing for a default number of mismatches (2 for the 1000 Genomes Project data set in our study which has read 100). Finally we compute the mean of the probabilities that the overlapped reads map to the target places where they are generated and use this as the mappability of the exon.

We compare our computed exonic mappability with the number of overlapped segmental duplications from the Segmental Duplication Database. Results show that not all segmental duplication regions are hard to map and thus it is not wise to directly filter out exons that overlap with segmental duplications (Supplementary Figure S2a). As a comparison, we also compute the sequence complexity--percentage of exon bases soft masked by RepeatMasker (http://www.repeatmasker.org/) using PLINK/SEQ (http://pngu.mgh.harvard.edu/purcell/plink/), which is the filtering strategy adopted by XHMM. It turns out that not only XHMM has an overly stringent threshold on sequence complexity/mappability (Supplementary Figure S2b), but also it includes other outlier removal steps, such as removing samples with coverage that are empirical outliers, filtering out targets with a standard deviation of PCA-normalized z-score greater than 30, etc. These additional empirical ways of excluding samples and targets might treat true signals as outliers and remove them.

**Supplementary Figure S1. Filtering strategies on mappability and sequence complexity by CODEX and XHMM.** Computation results from chromosome 22 are shown with filtering thresholds in dashed lines. (a) Mappability computed by CODEX. Exons that overlap with previously reported segmental duplications are marked in red. (b) Sequence complexity used in pre-filtering step by XHMM.

**Supplementary Figure S2. ROC curves of read depth normalization by CODEX and SVD-based method.** Gold standard is taken from the International HapMap Consortium SNP array CNV call set. The input for CODEX is the log_2_-ratio of the original read depth $Y$ versus the estimated control coverage $\widehat{\lambda}$; the input for SVD-based method is the residual obtained by subtracting the principal components from the original read depth $Y.$ For common CNVs shown in (a), (b), and (c), CODEX performs significantly better since SVD-based methods are optimized for rare CNV detection; for rare CNVs shown in (d) and (e), the two methods tend to have similar power for rare heterozygous deletions whereas CODEX performs better in detecting rare duplications. Of the 90 samples we analyze, there is no rare heterozygous deletion from the HapMap call set that we can use as a gold standard.


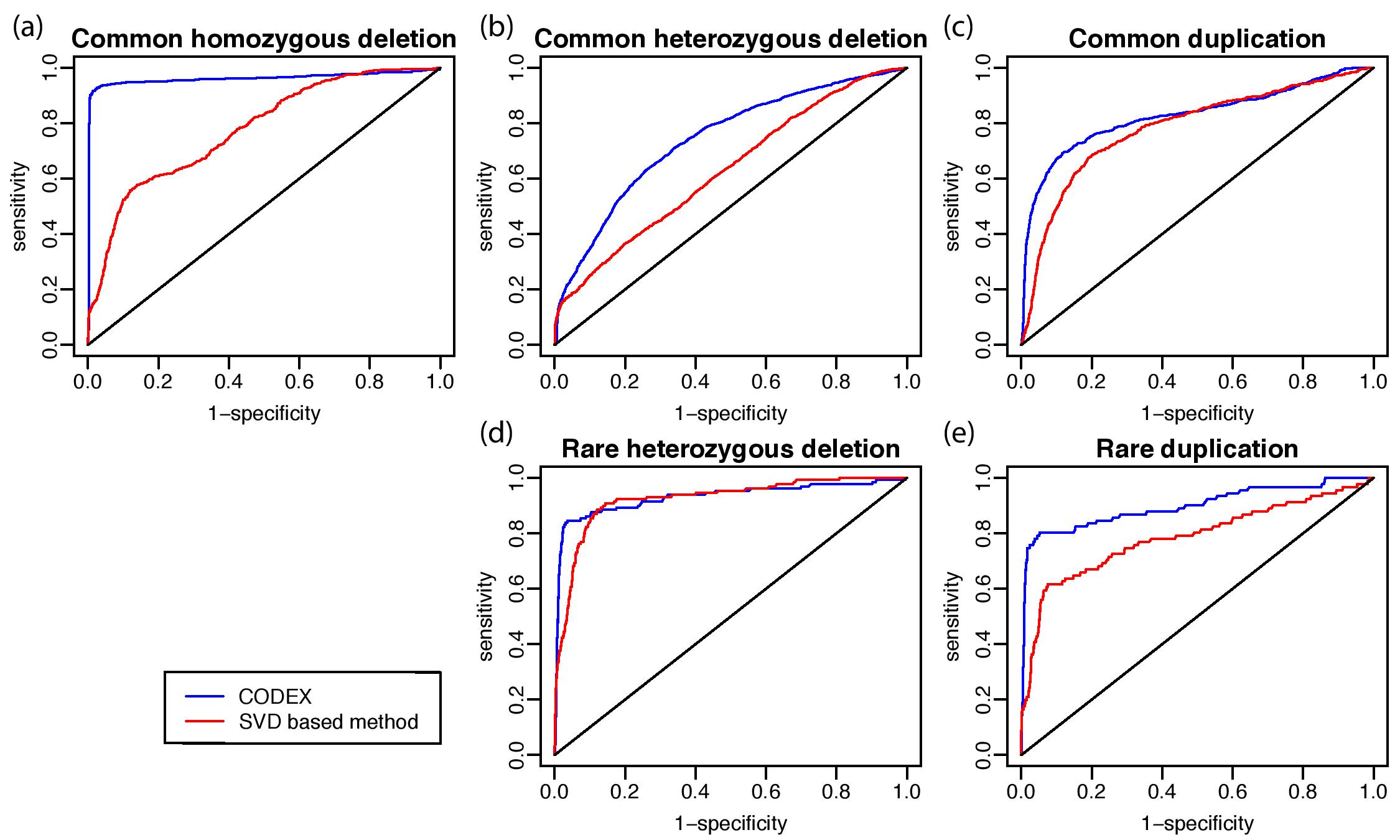


**Supplementary Figure S3. Scree plot from CoNIFER’s output on the 1000 Genomes Project WES data set.** Only plot for chromosome 1 is shown with the rest sharing similar if not exactly the same patterns. The variance reduction curve doesn’t level until the number of principal components reaches 7, indicating the choice of 4 is not too stringent.


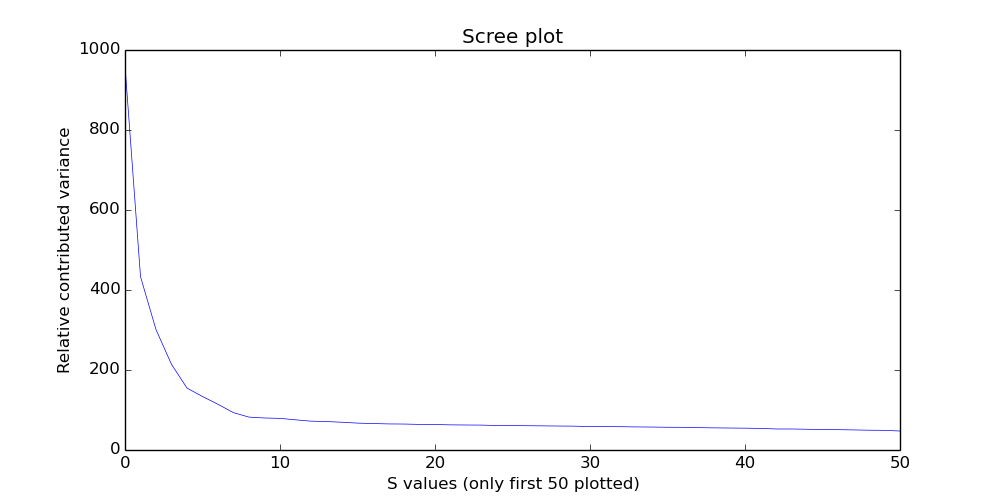


**Supplementary Figure S4. Spiked-in CNV signals for simulation studies.** (a) $\log(Y/\lambda)$ from CODEX of 10% spiked-in heterozygous deletions. (b) $\log(Y/\lambda)$ from CODEX of 90% spiked-in heterozygous deletions. It’s seen that the copy-number-neutral states have signals as duplications whereas deletions are normalized to be normal events.

**
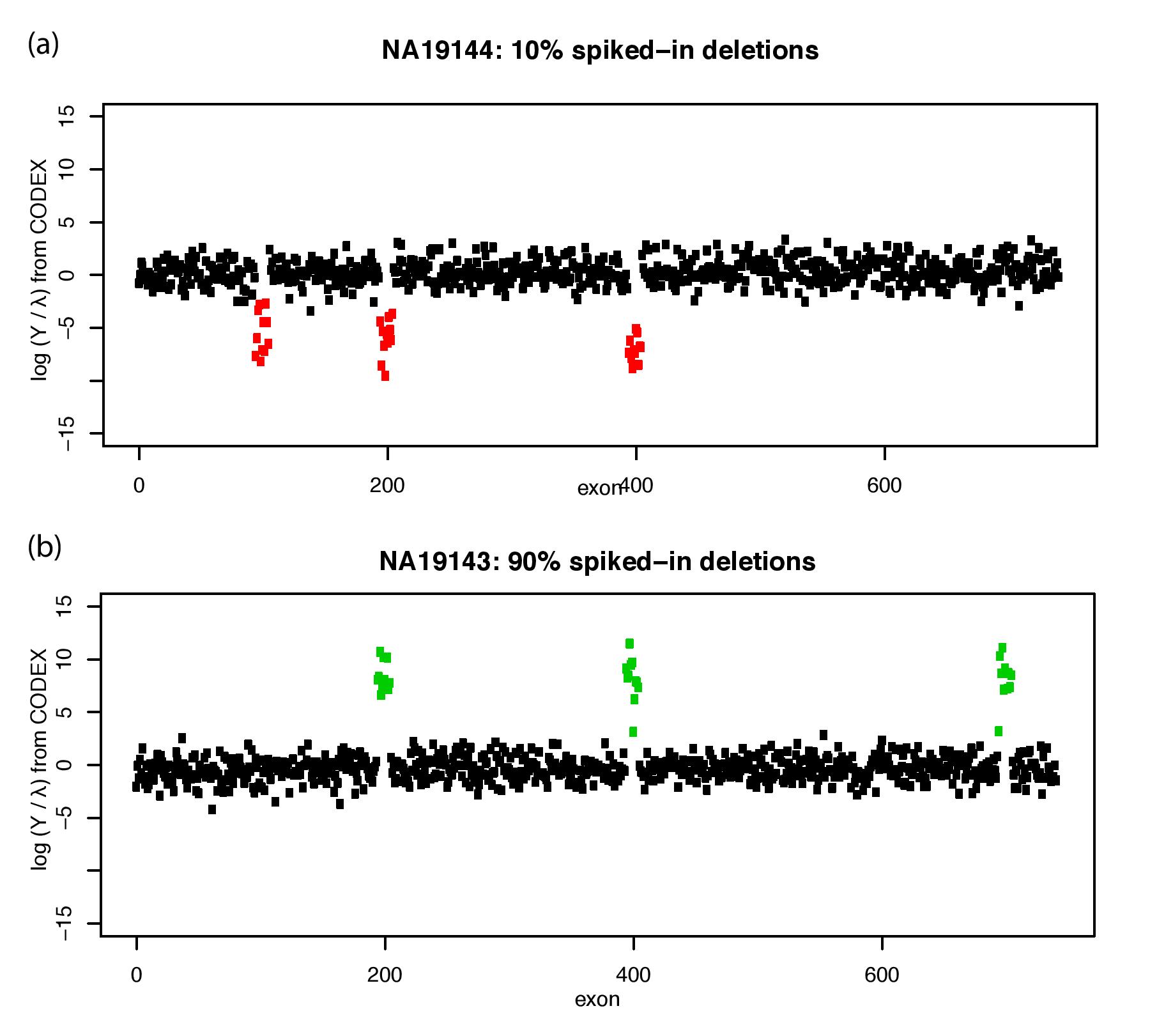
**

**Supplementary Figure S5. Power analysis of CODEX and SVD-based method, with and without QC procedures performed.** Simulated CNV events are of frequencies ranging from 5% to 95% and lengths (a) 5 exons and (b) 10 exons. Sensitivities of both methods decrease as CNV frequencies draw near 50%. Shorter CNV events are more often missed by the SVD approach whereas CODEX has comparable sensitivity for short and long variants at this scale. CODEX has higher power than SVD-based methods; QC procedures increase power for both methods by removing outliers.


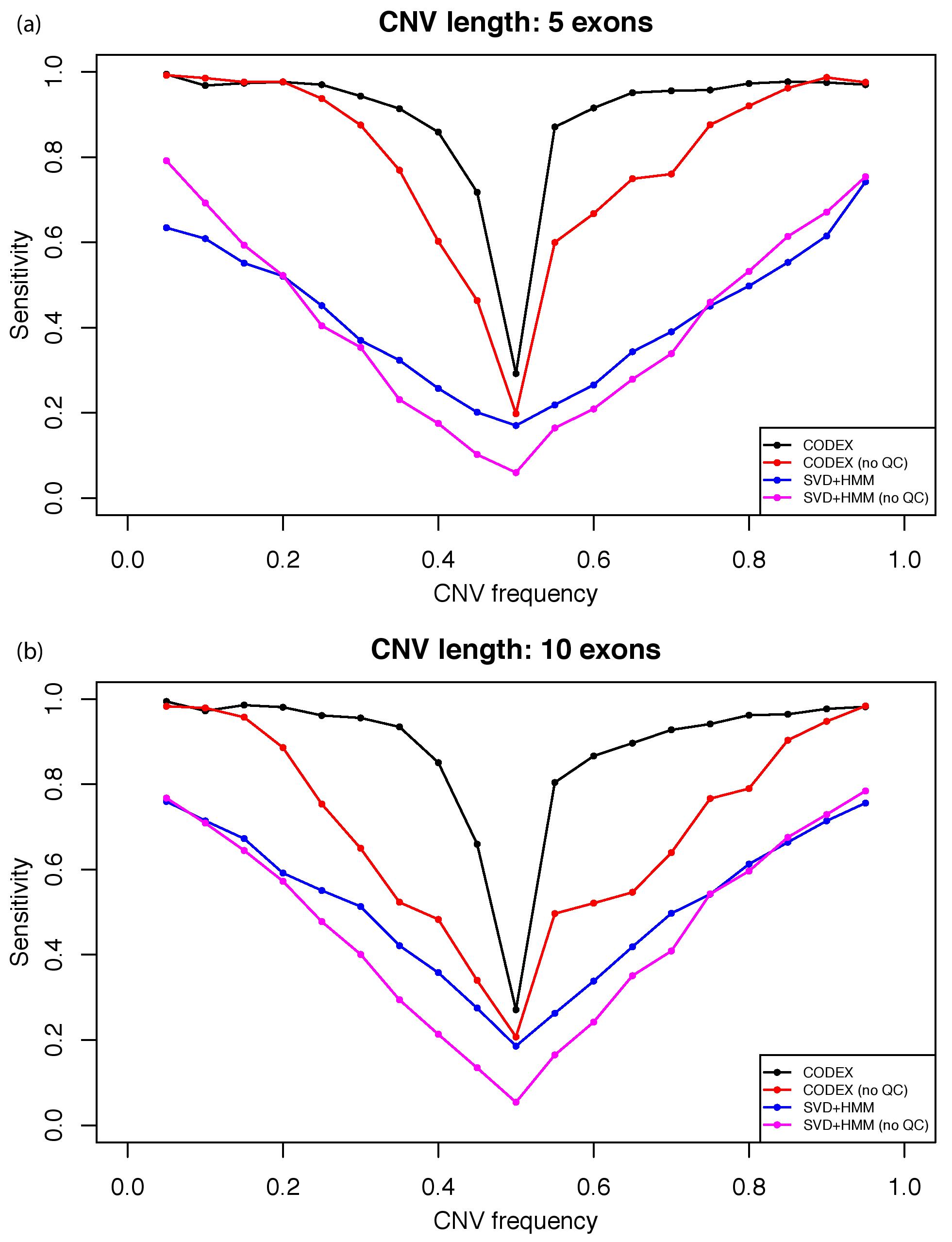


**Supplementary Figure S6. Correlation matrix plot of biases and artifacts shown in both exon-wise and sample-wise fashion.** $\beta,$ exon-wise latent factors, GC content, copy-number state, and pseudo-reference genome are interrogated in (a) and (c). Sample-wise latent factors, total number of reads per sample, sequencing centers, batch effects, and population are shown in (b) and (d). (a) and (b) are for spike-in CNV events with frequency 0.1 and (c) and (d) are for spike-in CNV events with frequency 0.4. $\beta$ and first exon-wise PC in SVD highly correlate with pseudo-reference genome. GC content is correlated with the third exon-wise PC in SVD with correlation coefficient -0.75. Copy-number states show higher correlation for spiked-in CNVs with higher frequencies. Sequencing centers and batch effects are captured by latent factors whereas population doesn’t seem to add too much variation to the CNV signals.


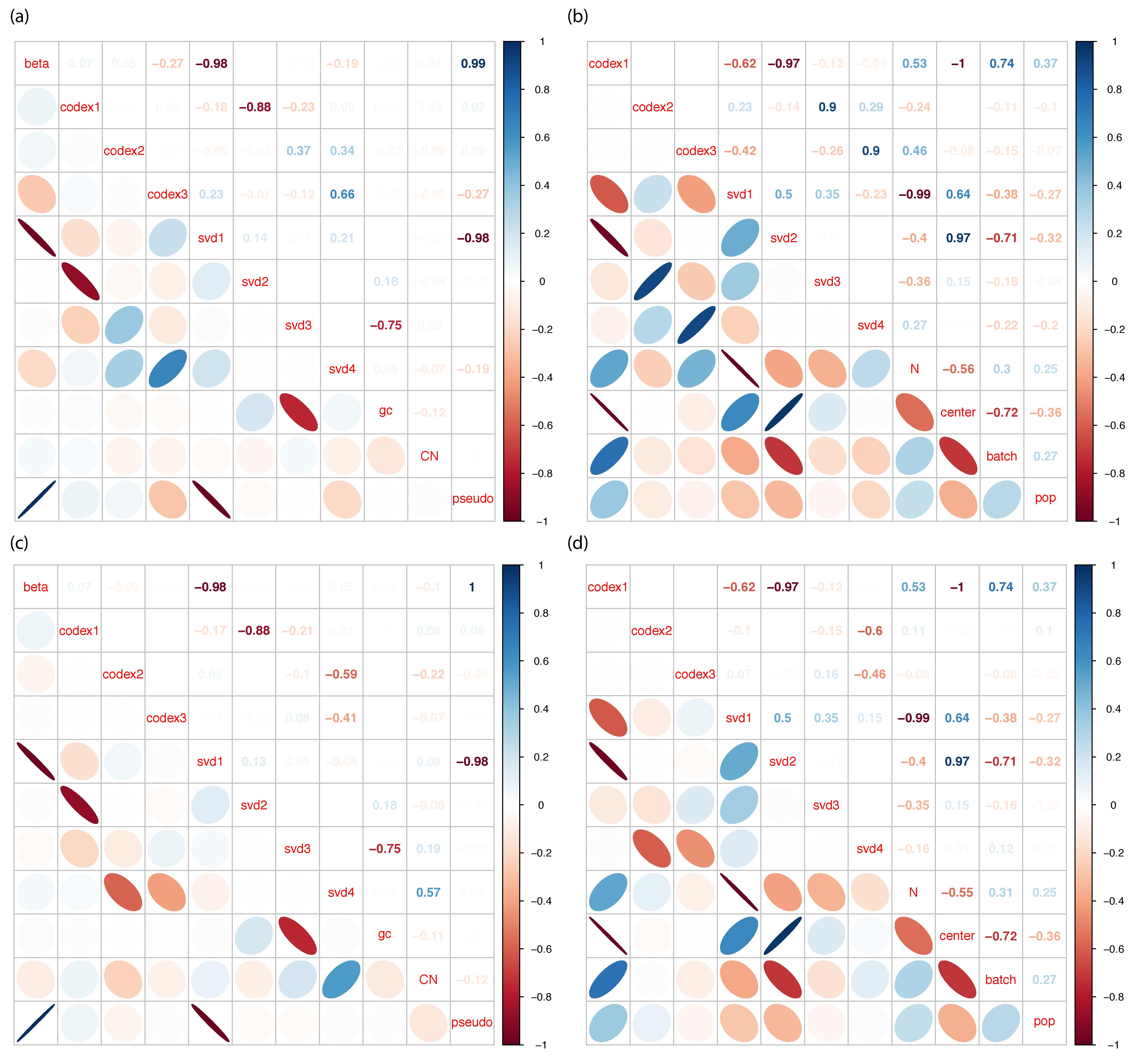


**Supplementary Figure S7. Zoom-in inspection of the deletions detected by CODEX in *ATRX* region.** All 18 samples detected are shown. Y-axis are $\log(Y/\lambda)$ of corresponding exons—values below zero indicate deletion events. The deletions called by CODEX are colored in red.
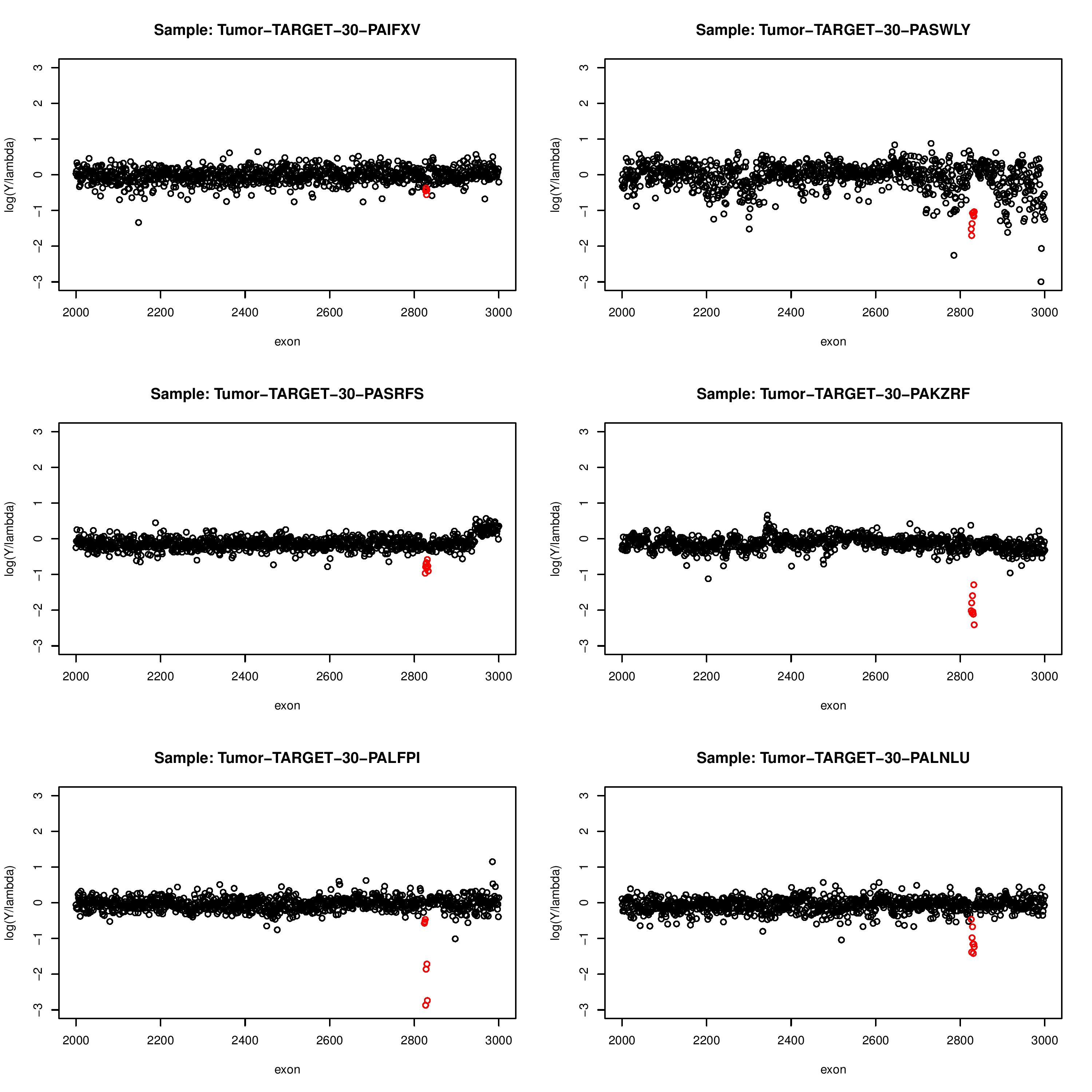


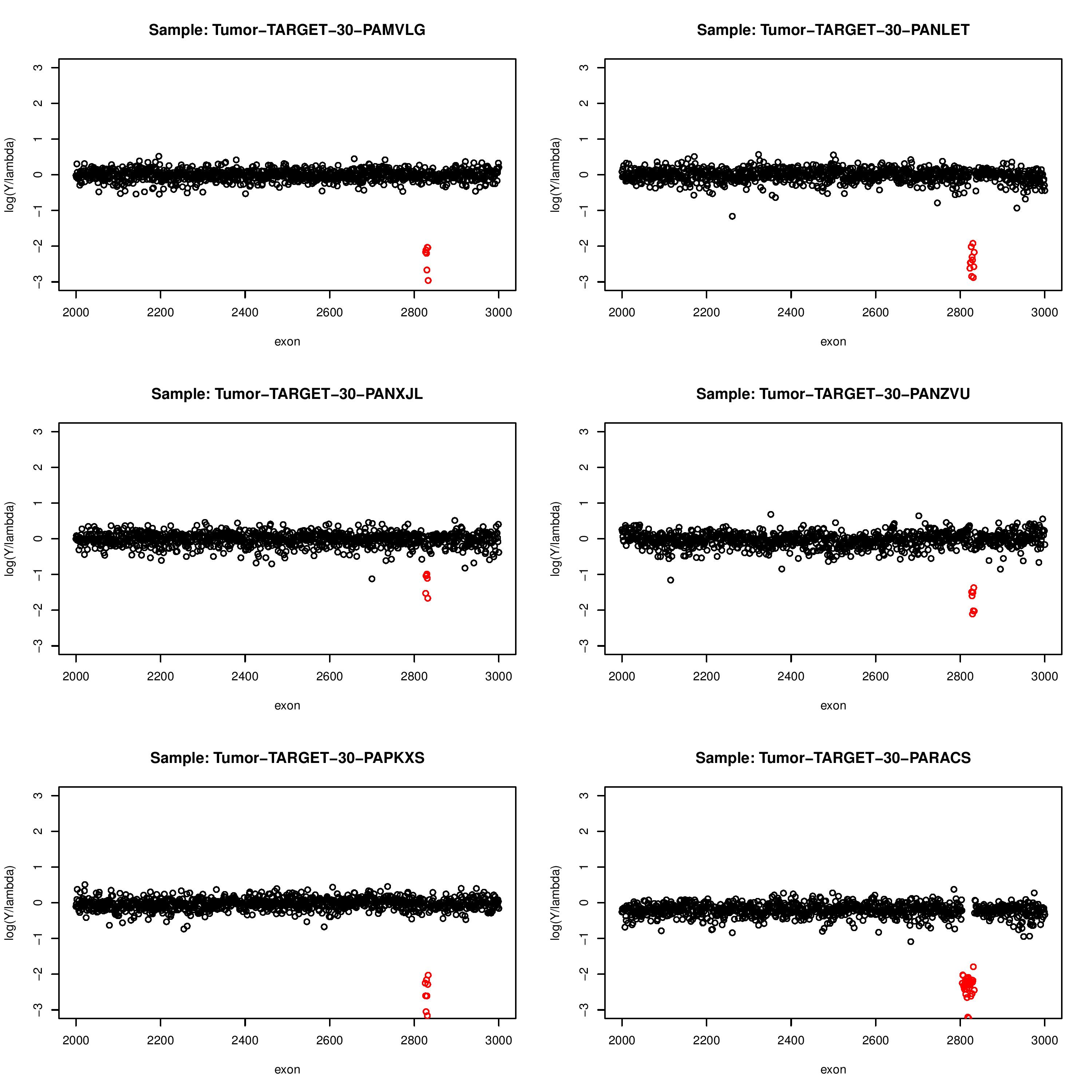


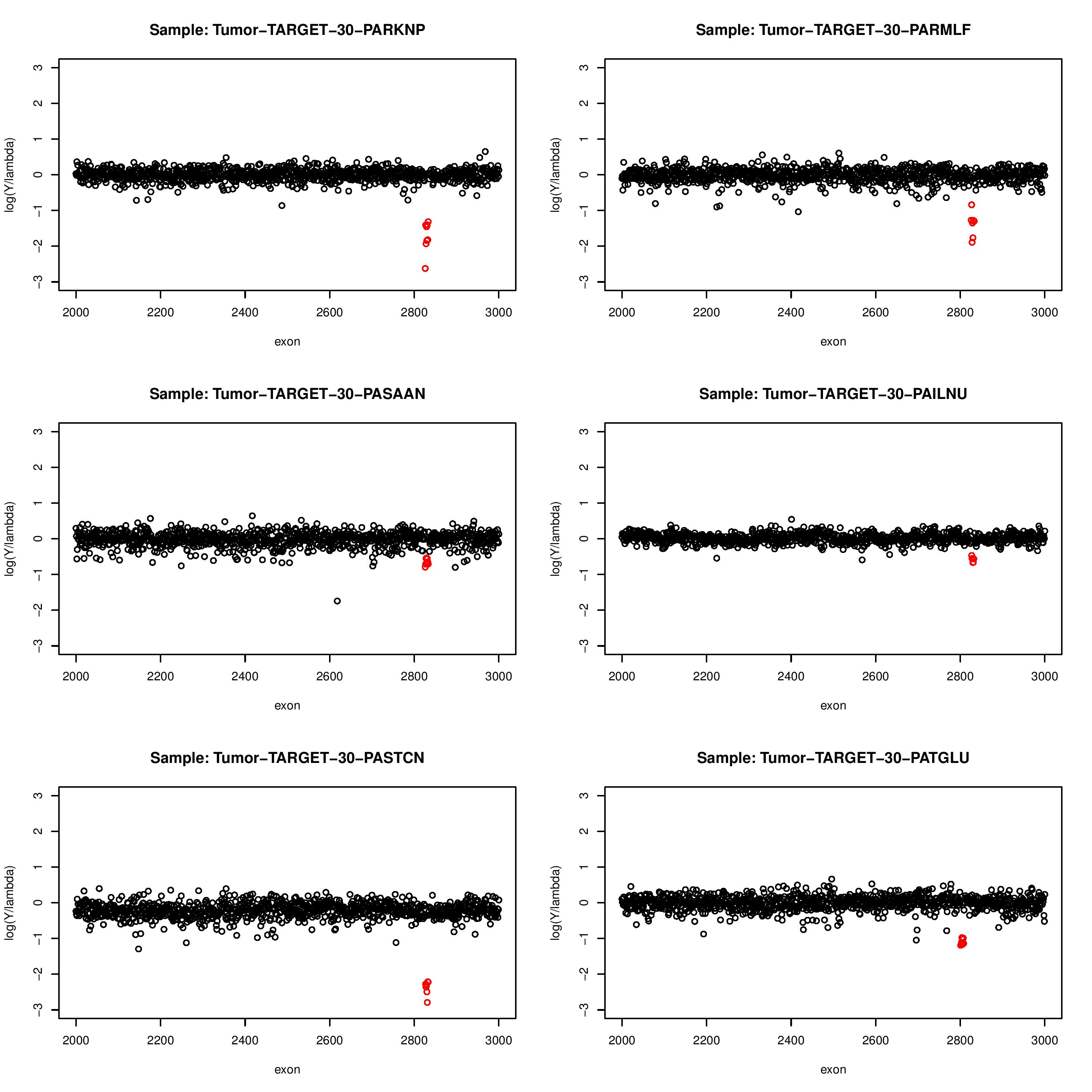


**Supplementary Table S1. Sample information of WES data set from the 1000 Genomes Project Phase 1 release.**

| **Sample ID** | **SRA individual sample accession number** | **Bam name** | **Read length** | **Population** | **Gender** | **Exome Center for Full Project** | **Has Omni Genotypes** | **Has Axiom Genotypes** | **Has more than 70% of Exome Targets covered to 20x or more** |
| --- | --- | --- | --- | --- | --- | --- | --- | --- | --- |
| NA06985 | SRS000030 | NA06985.mapped.ILLUMINA.bwa.CEU.exome.20130415.bam | 100 | CEU | female | BCM | 1 | 1 | 1 |
| NA07000 | SRS000033 | NA07000.mapped.ILLUMINA.bwa.CEU.exome.20130415.bam | 100 | CEU | female | BCM | 1 | 1 | 1 |
| NA07056 | SRS000594 | NA07056.mapped.ILLUMINA.bwa.CEU.exome.20130415.bam | 100 | CEU | female | BCM | 1 | 1 | 1 |
| NA07357 | SRS000038 | NA07357.mapped.ILLUMINA.bwa.CEU.exome.20130415.bam | 100 | CEU | male | BCM | 1 | 1 | 1 |
| NA10851* | SRS000040 | NA10851.mapped.ILLUMINA.bwa.CEU.exome.20130415.bam | 100 | CEU | male | BCM | 1 | 1 | 1 |
| NA11829 | SRS000041 | NA11829.mapped.ILLUMINA.bwa.CEU.exome.20130415.bam | 100 | CEU | male | BCM | 1 | 1 | 1 |
| NA11830 | SRS000042 | NA11830.mapped.ILLUMINA.bwa.CEU.exome.20130415.bam | 100 | CEU | female | BCM | 1 | 1 | 1 |
| NA11831 | SRS000043 | NA11831.mapped.ILLUMINA.bwa.CEU.exome.20130415.bam | 100 | CEU | male | BCM | 1 | 1 | 1 |
| NA11832 | SRS000044 | NA11832.mapped.ILLUMINA.bwa.CEU.exome.20130415.bam | 100 | CEU | female | BCM | 1 | 1 | 1 |
| NA11881 | SRS000046 | NA11881.mapped.ILLUMINA.bwa.CEU.exome.20130415.bam | 100 | CEU | male | BCM | 1 | 1 | 1 |
| NA11992 | SRS000052 | NA11992.mapped.ILLUMINA.bwa.CEU.exome.20130415.bam | 100 | CEU | male | BCM | 1 | 1 | 1 |
| NA11994 | SRS000054 | NA11994.mapped.ILLUMINA.bwa.CEU.exome.20130415.bam | 100 | CEU | male | BCM | 1 | 1 | 1 |
| NA11995 | SRS000055 | NA11995.mapped.ILLUMINA.bwa.CEU.exome.20130415.bam | 100 | CEU | female | BCM | 1 | 1 | 1 |
| NA12003 | SRS000056 | NA12003.mapped.ILLUMINA.bwa.CEU.exome.20130415.bam | 100 | CEU | male | BCM | 1 | 1 | 1 |
| NA12005 | SRS000058 | NA12005.mapped.ILLUMINA.bwa.CEU.exome.20130415.bam | 100 | CEU | male | BCM | 1 | 1 | 1 |
| NA12006 | SRS000059 | NA12006.mapped.ILLUMINA.bwa.CEU.exome.20130415.bam | 100 | CEU | female | BCM | 1 | 1 | 1 |
| NA12043 | SRS000060 | NA12043.mapped.ILLUMINA.bwa.CEU.exome.20130415.bam | 100 | CEU | male | BCM | 1 | 1 | 1 |
| NA12044 | SRS000061 | NA12044.mapped.ILLUMINA.bwa.CEU.exome.20130415.bam | 100 | CEU | female | BCM | 1 | 1 | 1 |
| NA12144 | SRS000063 | NA12144.mapped.ILLUMINA.bwa.CEU.exome.20130415.bam | 100 | CEU | male | BCM | 1 | 1 | 1 |
| NA12154 | SRS000064 | NA12154.mapped.ILLUMINA.bwa.CEU.exome.20130415.bam | 100 | CEU | male | BCM | 1 | 1 | 1 |
| NA12155 | SRS000065 | NA12155.mapped.ILLUMINA.bwa.CEU.exome.20130415.bam | 100 | CEU | male | BCM | 1 | 1 | 1 |
| NA12156 | SRS000066 | NA12156.mapped.ILLUMINA.bwa.CEU.exome.20130415.bam | 100 | CEU | female | BCM | 1 | 1 | 1 |
| NA12234 | SRS000067 | NA12234.mapped.ILLUMINA.bwa.CEU.exome.20130415.bam | 100 | CEU | female | BCM | 1 | 1 | 1 |
| NA12762 | SRS000079 | NA12762.mapped.ILLUMINA.bwa.CEU.exome.20130415.bam | 100 | CEU | male | BCM | 1 | 1 | 1 |
| NA12812 | SRS000082 | NA12812.mapped.ILLUMINA.bwa.CEU.exome.20130415.bam | 100 | CEU | male | BCM | 1 | 1 | 1 |
| NA12813 | SRS000083 | NA12813.mapped.ILLUMINA.bwa.CEU.exome.20130415.bam | 100 | CEU | female | BCM | 1 | 1 | 1 |
| NA12814 | SRS000084 | NA12814.mapped.ILLUMINA.bwa.CEU.exome.20130415.bam | 100 | CEU | male | BCM | 1 | 1 | 1 |
| NA12815 | SRS000085 | NA12815.mapped.ILLUMINA.bwa.CEU.exome.20130415.bam | 100 | CEU | female | BCM | 1 | 1 | 1 |
| NA12872 | SRS000087 | NA12872.mapped.ILLUMINA.bwa.CEU.exome.20130415.bam | 100 | CEU | male | BCM | 1 | 1 | 1 |
| NA12873 | SRS000088 | NA12873.mapped.ILLUMINA.bwa.CEU.exome.20130415.bam | 100 | CEU | female | BCM | 1 | 1 | 1 |
| NA12874 | SRS000089 | NA12874.mapped.ILLUMINA.bwa.CEU.exome.20130415.bam | 100 | CEU | male | BCM | 1 | 1 | 1 |
| NA18502* | SRS000098 | NA18502.mapped.ILLUMINA.bwa.YRI.exome.20130415.bam | 100 | YRI | female | BCM | 1 | 1 | 1 |
| NA18505 | SRS000100 | NA18505.mapped.ILLUMINA.bwa.YRI.exome.20130415.bam | 100 | YRI | female | BCM | 1 | 1 | 1 |
| NA18507 | SRS000101 | NA18507.mapped.ILLUMINA.bwa.YRI.exome.20130415.bam | 100 | YRI | male | BCM | 1 | 1 | 1 |
| NA18508 | SRS000102 | NA18508.mapped.ILLUMINA.bwa.YRI.exome.20130415.bam | 100 | YRI | female | BCM | 1 | 1 | 1 |
| NA18956 | SRS000162 | NA18956.mapped.ILLUMINA.bwa.JPT.exome.20130415.bam | 100 | JPT | female | BCM | 1 | 1 | 1 |
| NA18965 | SRS000167 | NA18965.mapped.ILLUMINA.bwa.JPT.exome.20130415.bam | 100 | JPT | male | BCM | 1 | 1 | 1 |
| NA18978 | SRS000725 | NA18978.mapped.ILLUMINA.bwa.JPT.exome.20130415.bam | 100 | JPT | female | BCM | 1 | 1 | 1 |
| NA18980 | SRS000178 | NA18980.mapped.ILLUMINA.bwa.JPT.exome.20130415.bam | 100 | JPT | female | BCM | 1 | 1 | 1 |
| NA19005 | SRS000180 | NA19005.mapped.ILLUMINA.bwa.JPT.exome.20130415.bam | 100 | JPT | male | BCM | 1 | 1 | 1 |
| NA19099 | SRS000183 | NA19099.mapped.ILLUMINA.bwa.YRI.exome.20130422.bam | 100 | YRI | female | BCM | 1 | 1 | 1 |
| NA19222 | SRS001802 | NA19222.mapped.ILLUMINA.bwa.YRI.exome.20130422.bam | 100 | YRI | female | BCM | 1 | 1 | 1 |
| NA18994 | SRS000732 | NA18994.mapped.ILLUMINA.bwa.JPT.exome.20130415.bam | 100 | JPT | male | BCM | 1 | 1 | 1 |
| NA18995 | SRS000733 | NA18995.mapped.ILLUMINA.bwa.JPT.exome.20130415.bam | 100 | JPT | male | BCM | 1 | 1 | 1 |
| NA18997 | SRS000734 | NA18997.mapped.ILLUMINA.bwa.JPT.exome.20130415.bam | 100 | JPT | female | BCM | 1 | 1 | 1 |
| NA18998 | SRS000735 | NA18998.mapped.ILLUMINA.bwa.JPT.exome.20130415.bam | 100 | JPT | female | BCM | 1 | 1 | 1 |
| NA06994 | SRS000032 | NA06994.mapped.ILLUMINA.bwa.CEU.exome.20120522.bam | 100 | CEU | male | WUGSC | 1 | 1 | 1 |
| NA10847 | SRS000039 | NA10847.mapped.ILLUMINA.bwa.CEU.exome.20121211.bam | 100 | CEU | female | WUGSC | 1 | 1 | 1 |
| NA11840 | SRS000045 | NA11840.mapped.ILLUMINA.bwa.CEU.exome.20120522.bam | 100 | CEU | female | WUGSC | 1 | 1 | 1 |
| NA12249 | SRS000068 | NA12249.mapped.ILLUMINA.bwa.CEU.exome.20120522.bam | 100 | CEU | female | WUGSC | 1 | 1 | 1 |
| NA12272* | SRS000616 | NA12272.mapped.ILLUMINA.bwa.CEU.exome.20120522.bam | 100 | CEU | male | WUGSC | 1 | 1 | 1 |
| NA12716 | SRS000072 | NA12716.mapped.ILLUMINA.bwa.CEU.exome.20121211.bam | 100 | CEU | male | WUGSC | 1 | 1 | 1 |
| NA12750 | SRS000075 | NA12750.mapped.ILLUMINA.bwa.CEU.exome.20130415.bam | 100 | CEU | male | WUGSC | 1 | 1 | 1 |
| NA12751 | SRS000076 | NA12751.mapped.ILLUMINA.bwa.CEU.exome.20121211.bam | 100 | CEU | female | WUGSC | 1 | 1 | 1 |
| NA12760 | SRS000077 | NA12760.mapped.ILLUMINA.bwa.CEU.exome.20120522.bam | 100 | CEU | male | WUGSC | 1 | 1 | 1 |
| NA12761 | SRS000078 | NA12761.mapped.ILLUMINA.bwa.CEU.exome.20121211.bam | 100 | CEU | female | WUGSC | 1 | 1 | 1 |
| NA12763 | SRS000080 | NA12763.mapped.ILLUMINA.bwa.CEU.exome.20121211.bam | 100 | CEU | female | WUGSC | 1 | 1 | 1 |
| NA18966 | SRS000723 | NA18966.mapped.ILLUMINA.bwa.JPT.exome.20121211.bam | 100 | JPT | male | WUGSC | 1 | 1 | 1 |
| NA18967 | SRS000168 | NA18967.mapped.ILLUMINA.bwa.JPT.exome.20121211.bam | 100 | JPT | male | WUGSC | 1 | 1 | 1 |
| NA18968 | SRS000169 | NA18968.mapped.ILLUMINA.bwa.JPT.exome.20120522.bam | 100 | JPT | female | WUGSC | 1 | 1 | 1 |
| NA18969 | SRS000170 | NA18969.mapped.ILLUMINA.bwa.JPT.exome.20120522.bam | 100 | JPT | female | WUGSC | 1 | 1 | 1 |
| NA18970 | SRS000171 | NA18970.mapped.ILLUMINA.bwa.JPT.exome.20120522.bam | 100 | JPT | male | WUGSC | 1 | 1 | 1 |
| NA18971 | SRS000172 | NA18971.mapped.ILLUMINA.bwa.JPT.exome.20120522.bam | 100 | JPT | male | WUGSC | 1 | 1 | 1 |
| NA18972 | SRS000173 | NA18972.mapped.ILLUMINA.bwa.JPT.exome.20121211.bam | 100 | JPT | female | WUGSC | 1 | 1 | 1 |
| NA18973 | SRS000174 | NA18973.mapped.ILLUMINA.bwa.JPT.exome.20121211.bam | 100 | JPT | female | WUGSC | 1 | 1 | 1 |
| NA18974 | SRS000175 | NA18974.mapped.ILLUMINA.bwa.JPT.exome.20120522.bam | 100 | JPT | male | WUGSC | 1 | 1 | 1 |
| NA18975 | SRS000176 | NA18975.mapped.ILLUMINA.bwa.JPT.exome.20120522.bam | 100 | JPT | female | WUGSC | 1 | 1 | 1 |
| NA18976 | SRS000177 | NA18976.mapped.ILLUMINA.bwa.JPT.exome.20120522.bam | 100 | JPT | female | WUGSC | 1 | 1 | 1 |
| NA18981 | SRS000179 | NA18981.mapped.ILLUMINA.bwa.JPT.exome.20120522.bam | 100 | JPT | female | WUGSC | 1 | 1 | 1 |
| NA18987 | SRS000727 | NA18987.mapped.ILLUMINA.bwa.JPT.exome.20120522.bam | 100 | JPT | female | WUGSC | 1 | 1 | 1 |
| NA18990 | SRS000728 | NA18990.mapped.ILLUMINA.bwa.JPT.exome.20120522.bam | 100 | JPT | male | WUGSC | 1 | 1 | 1 |
| NA18991 | SRS000729 | NA18991.mapped.ILLUMINA.bwa.JPT.exome.20120522.bam | 100 | JPT | female | WUGSC | 1 | 1 | 1 |
| NA19072* | SRS000760 | NA19072.mapped.ILLUMINA.bwa.JPT.exome.20120522.bam | 100 | JPT | male | WUGSC | 1 | 1 | 1 |
| NA19098 | SRS000182 | NA19098.mapped.ILLUMINA.bwa.YRI.exome.20121211.bam | 100 | YRI | male | WUGSC | 1 | 1 | 1 |
| NA19119 | SRS000188 | NA19119.mapped.ILLUMINA.bwa.YRI.exome.20120522.bam | 100 | YRI | male | WUGSC | 1 | 1 | 1 |
| NA19131 | SRS000190 | NA19131.mapped.ILLUMINA.bwa.YRI.exome.20120522.bam | 100 | YRI | female | WUGSC | 1 | 1 | 1 |
| NA19137 | SRS000191 | NA19137.mapped.ILLUMINA.bwa.YRI.exome.20130415.bam | 100 | YRI | female | WUGSC | 1 | 1 | 1 |
| NA19138 | SRS000192 | NA19138.mapped.ILLUMINA.bwa.YRI.exome.20121211.bam | 100 | YRI | male | WUGSC | 1 | 1 | 1 |
| NA19141 | SRS000193 | NA19141.mapped.ILLUMINA.bwa.YRI.exome.20120522.bam | 100 | YRI | male | WUGSC | 1 | 1 | 1 |
| NA19143 | SRS000194 | NA19143.mapped.ILLUMINA.bwa.YRI.exome.20120522.bam | 100 | YRI | female | WUGSC | 1 | 1 | 1 |
| NA19144 | SRS000195 | NA19144.mapped.ILLUMINA.bwa.YRI.exome.20121211.bam | 100 | YRI | male | WUGSC | 1 | 1 | 1 |
| NA19152 | SRS000197 | NA19152.mapped.ILLUMINA.bwa.YRI.exome.20120522.bam | 100 | YRI | female | WUGSC | 1 | 1 | 1 |
| NA19153 | SRS000198 | NA19153.mapped.ILLUMINA.bwa.YRI.exome.20121211.bam | 100 | YRI | male | WUGSC | 1 | 1 | 1 |
| NA19159 | SRS000199 | NA19159.mapped.ILLUMINA.bwa.YRI.exome.20121211.bam | 100 | YRI | female | WUGSC | 1 | 1 | 1 |
| NA19160 | SRS000200 | NA19160.mapped.ILLUMINA.bwa.YRI.exome.20120522.bam | 100 | YRI | male | WUGSC | 1 | 1 | 1 |
| NA19171 | SRS000201 | NA19171.mapped.ILLUMINA.bwa.YRI.exome.20120522.bam | 100 | YRI | male | WUGSC | 1 | 1 | 1 |
| NA19200 | SRS000204 | NA19200.mapped.ILLUMINA.bwa.YRI.exome.20120522.bam | 100 | YRI | male | WUGSC | 1 | 1 | 1 |
| NA19201 | SRS000205 | NA19201.mapped.ILLUMINA.bwa.YRI.exome.20121211.bam | 100 | YRI | female | WUGSC | 1 | 1 | 1 |
| NA19204 | SRS000206 | NA19204.mapped.ILLUMINA.bwa.YRI.exome.20120522.bam | 100 | YRI | female | WUGSC | 1 | 1 | 1 |
| NA19206 | SRS000207 | NA19206.mapped.ILLUMINA.bwa.YRI.exome.20121211.bam | 100 | YRI | female | WUGSC | 1 | 1 | 1 |
| NA19207 | SRS000208 | NA19207.mapped.ILLUMINA.bwa.YRI.exome.20121211.bam | 100 | YRI | male | WUGSC | 1 | 1 | 1 |
| NA19209 | SRS000209 | NA19209.mapped.ILLUMINA.bwa.YRI.exome.20121211.bam | 100 | YRI | female | WUGSC | 1 | 1 | 1 |
| NA19210 | SRS000210 | NA19210.mapped.ILLUMINA.bwa.YRI.exome.20121211.bam | 100 | YRI | male | WUGSC | 1 | 1 | 1 |
| NA19223 | SRS000800 | NA19223.mapped.ILLUMINA.bwa.YRI.exome.20120522.bam | 100 | YRI | male | WUGSC | 1 | 1 | 1 |

| More detailed information: ftp://ftp.1000genomes.ebi.ac.uk/vol1/ftp/technical/working/20111108_samples_pedigree/20111108_1000genomes_samples.xls. |
| --- |
| *Samples only used as controls by EXCAVATOR. |

**Supplementary Table S2.** **1000 Genomes Project WES data set CNV calls by CODEX, XHMM, CoNIFER, and EXCAVATOR.**

**Supplementary Table S3. SNP array validation data sets on the 1000 Genomes Project WES data set from the International HapMap Consortium, Conrad *et al.*, and McCarroll *et al*.**

**Supplementary Table S4. Sensitivity, specificity, and precision rate of CNV calls by CODEX, XHMM, CoNIFER, and EXCAVATOR.** The plot of precision and recall rates are shown in Figure 5. Three “gold-standard” CNV metrics are adopted from (a) International HapMap Consortium, (b) Conrad *et al.*, and (c) McCarroll *et al..* CODEX and XHMM performs better in detecting rare CNVs compared to common ones, with CODEX having the highest F-measure among all methods compared.

| (a) |  |  |  |  |  |  |  |  |  |  |  |  |  |  |  |  |  |  |  |  |  |
| --- | --- | --- | --- | --- | --- | --- | --- | --- | --- | --- | --- | --- | --- | --- | --- | --- | --- | --- | --- | --- | --- |
| **Software** | **Sensitivity/specificity/precision/TP/FP/TN/FN compared to HapMap3 callset** | | | | | | | | | | | | | | | | | | | | |
|  | **All** | | | | | | | **Common** | | | | | | | **Rare** | | | | | | |
| CODEX | 0.22 | 0.95 | 0.63 | 377 | 225 | 4551 | 1309 | 0.22 | 0.93 | 0.65 | 363 | 198 | 2644 | 1284 | 0.36 | 0.99 | 0.34 | 14 | 27 | 1907 | 25 |
| XHMM | 0.05 | 0.98 | 0.49 | 82 | 84 | 4638 | 1658 | 0.04 | 0.98 | 0.61 | 70 | 45 | 2743 | 1631 | 0.31 | 0.98 | 0.24 | 12 | 39 | 1895 | 27 |
| CoNIFER | 0.01 | 1.00 | 0.77 | 10 | 3 | 4712 | 1737 | 0.01 | 1.00 | 0.77 | 10 | 3 | 2778 | 1698 | 0.00 | 1.00 | NaN | 0 | 0 | 1934 | 39 |
| EXCAVATOR | 0.14 | 0.92 | 0.37 | 222 | 386 | 4433 | 1421 | 0.14 | 0.89 | 0.40 | 218 | 322 | 2563 | 1386 | 0.10 | 0.97 | 0.06 | 4 | 64 | 1870 | 35 |
|  |  |  |  |  |  |  |  |  |  |  |  |  |  |  |  |  |  |  |  |  |  |
| (b) |  |  |  |  |  |  |  |  |  |  |  |  |  |  |  |  |  |  |  |  |  |
| **Software** | **Sensitivity/specificity/precision/TP/FP/TN/FN compared to Conrad *et al.* callset** | | | | | | | | | | | | | | | | | | | | |
|  | **All** | | | | | | | **Common** | | | | | | | **Rare** | | | | | | |
| CODEX | 0.08 | 0.99 | 0.56 | 493 | 383 | 26892 | 5378 | 0.08 | 0.95 | 0.62 | 449 | 278 | 4993 | 5195 | 0.19 | 1.00 | 0.30 | 44 | 105 | 21899 | 183 |
| XHMM | 0.03 | 0.99 | 0.52 | 180 | 169 | 27064 | 5733 | 0.03 | 0.99 | 0.65 | 144 | 77 | 5152 | 5542 | 0.16 | 1.00 | 0.28 | 36 | 92 | 21912 | 191 |
| CoNIFER | 0.00 | 1.00 | 0.16 | 3 | 16 | 27187 | 5940 | 0.00 | 1.00 | 0.15 | 2 | 11 | 5188 | 5714 | 0.00 | 1.00 | 0.17 | 1 | 5 | 21999 | 226 |
| EXCAVATOR | 0.07 | 0.98 | 0.39 | 424 | 668 | 26603 | 5451 | 0.07 | 0.93 | 0.53 | 402 | 357 | 4909 | 5247 | 0.10 | 0.99 | 0.07 | 22 | 311 | 21694 | 204 |
|  |  |  |  |  |  |  |  |  |  |  |  |  |  |  |  |  |  |  |  |  |  |
| (c) |  |  |  |  |  |  |  |  |  |  |  |  |  |  |  |  |  |  |  |  |  |
| **Software** | **Sensitivity/specificity/precision/TP/FP/TN/FN compared to McCarroll *et al.* callset** | | | | | | | | | | | | | | | | | | | | |
|  | **All** | | | | | | | **Common** | | | | | | | **Rare** | | | | | | |
| CODEX | 0.17 | 0.97 | 0.67 | 480 | 239 | 8404 | 2306 | 0.16 | 0.94 | 0.67 | 436 | 215 | 3419 | 2274 | 0.58 | 1.00 | 0.65 | 44 | 24 | 4985 | 32 |
| XHMM | 0.07 | 0.99 | 0.61 | 196 | 123 | 8489 | 2621 | 0.06 | 0.97 | 0.63 | 155 | 92 | 3511 | 2586 | 0.54 | 0.99 | 0.57 | 41 | 31 | 4978 | 35 |
| CoNIFER | 0.01 | 1.00 | 0.79 | 19 | 5 | 8580 | 2825 | 0.01 | 1.00 | 0.83 | 15 | 3 | 3573 | 2753 | 0.05 | 1.00 | 0.67 | 4 | 2 | 5007 | 72 |
| EXCAVATOR | 0.12 | 0.94 | 0.40 | 329 | 497 | 8205 | 2398 | 0.12 | 0.89 | 0.44 | 311 | 394 | 3299 | 2340 | 0.24 | 0.98 | 0.15 | 18 | 103 | 4906 | 58 |

**Supplementary Table S5. Somatic deletions within *ATRX* region detected using WES data of neuroblastoma patients.** (a) Summary of deletions detected by tumor/normal threshold, CODEX, and XHMM with break-point and length information. Of the 18 samples detected by CODEX, 16 samples overlap with the matched tumor blood analysis result; 14 and all of XHMM’s CNV events are detected; one sample is uniquely called. Breakpoints may differ slightly between different methods but are within reasonable limits. (b) Deletions detected by thresholdhing log_2_-ratio of tumor RPKM to blood RPKM. (c) Deletions detected using tumor samples only by CODEX. (d) Deletions detected using tumor samples only by XHMM.

(a)

| **Sample** | **Pugh *et al.* (tumor/normal)** | | | **CODEX** | | | **XHMM** | | |
| --- | --- | --- | --- | --- | --- | --- | --- | --- | --- |
|  | **Start** | **End** | **Length (Kb)** | **Start** | **End** | **Length (Kb)** | **Start** | **End** | **Length (Kb)** |
| TARGET.30.PAIFXV |  |  |  | 76937010 | 76952194 | 15.185 |  |  |  |
| TARGET.30.PASWLY |  |  |  | 76931719 | 76972722 | 41.004 | 76931719 | 76972722 | 41.004 |
| TARGET.30.PASRFS | 76931795 | 76972722 | 40.928 | 76931719 | 76972722 | 41.004 |  |  |  |
| TARGET.30.PAKZRF | 76931795 | 76972722 | 40.928 | 76931719 | 76972722 | 41.004 |  |  |  |
| TARGET.30.PALFPI | 76940087 | 76953125 | 13.039 | 76918869 | 76954119 | 35.251 |  |  |  |
| TARGET.30.PALNLU | 76940087 | 76972722 | 32.636 | 76937010 | 76972722 | 35.713 | 76931719 | 76972722 | 41.004 |
| TARGET.30.PAMVLG | 76931795 | 76972722 | 40.928 | 76937010 | 76972722 | 35.713 | 76937010 | 76972722 | 35.713 |
| TARGET.30.PANLET | 76919049 | 76953125 | 34.077 | 76912048 | 76972722 | 60.675 | 76912048 | 76972722 | 60.675 |
| TARGET.30.PANXJL | 76940087 | 76953125 | 13.039 | 76937010 | 76954119 | 17.11 | 76937010 | 76954119 | 17.11 |
| TARGET.30.PANZVU | 76940500 | 76972722 | 32.223 | 76931719 | 76972722 | 41.004 | 76937010 | 76972722 | 35.713 |
| TARGET.30.PAPKXS | 76931795 | 76953125 | 21.331 | 76931719 | 76972722 | 41.004 | 76931719 | 76972722 | 41.004 |
| TARGET.30.PARACS | 76778881 | 76972722 | 193.842 | 76778728 | 76972722 | 193.995 | 76778728 | 76972722 | 193.995 |
| TARGET.30.PARKNP | 76931795 | 76972722 | 40.928 | 76931719 | 76972722 | 41.004 | 76931719 | 76972722 | 41.004 |
| TARGET.30.PARMLF | 76931795 | 76972722 | 40.928 | 76931719 | 76972722 | 41.004 | 76931719 | 76972722 | 41.004 |
| TARGET.30.PASAAN | 76931795 | 76972722 | 40.928 | 76931719 | 76972722 | 41.004 | 76931719 | 76972722 | 41.004 |
| TARGET.30.PAILNU | 76940087 | 76953125 | 13.039 | 76937010 | 76954119 | 17.11 | 76931719 | 76954119 | 22.401 |
| TARGET.30.PASTCN | 76940087 | 76972722 | 32.636 | 76937010 | 76972722 | 35.713 | 76937010 | 76972722 | 35.713 |
| TARGET.30.PATGLU | 76764109 | 76845412 | 81.304 | 76763827 | 76829825 | 65.999 | 76763827 | 76829825 | 65.999 |

(b)

| **Sample** | **Chromosome** | **Start** | **End** | **Length (Kb)** | **Num_Probes** | **Segment_Mean** |
| --- | --- | --- | --- | --- | --- | --- |
| TARGET.30.PAMVLG | X | 76931795 | 76940500 | 8.706 | 3 | -2.53655 |
| TARGET.30.PAMVLG | X | 76944422 | 76972722 | 28.301 | 5 | -26.10105 |
| TARGET.30.PANLET | X | 76919049 | 76953125 | 34.077 | 9 | -25.48575 |
| TARGET.30.PANXJL | X | 76940087 | 76953125 | 13.039 | 6 | -1.8623 |
| TARGET.30.PAPKXS | X | 76931795 | 76953125 | 21.331 | 7 | -23.9693 |
| TARGET.30.PARACS | X | 76778881 | 76814319 | 35.439 | 3 | -2.7718 |
| TARGET.30.PARACS | X | 76829825 | 76949428 | 119.604 | 22 | -19.7517 |
| TARGET.30.PARACS | X | 76952194 | 76972722 | 20.529 | 3 | -2.9612 |
| TARGET.30.PARKNP | X | 76931795 | 76944422 | 12.628 | 4 | -17.2465 |
| TARGET.30.PARKNP | X | 76949428 | 76972722 | 23.295 | 4 | -2.9277 |
| TARGET.30.PARMLF | X | 76931795 | 76949428 | 17.634 | 5 | -13.7373 |
| TARGET.30.PARMLF | X | 76952194 | 76972722 | 20.529 | 3 | -2.6691 |
| TARGET.30.PASAAN | X | 76931795 | 76972722 | 40.928 | 8 | -1.06985 |
| TARGET.30.PASRFS | X | 76931795 | 76972722 | 40.928 | 8 | -1.0058 |
| TARGET.30.PASTCN | X | 76940087 | 76972722 | 32.636 | 7 | -2.689 |
| TARGET.30.PATGLU | X | 76764109 | 76845412 | 81.304 | 9 | -1.5369 |
| TARGET-30-PAILNU | X | 76940087 | 76953125 | 13.039 | 6 | -0.905 |
| TARGET-30-PAKZRF | X | 76931795 | 76972722 | 40.928 | 8 | -2.6115 |
| TARGET-30-PALFPI | X | 76940087 | 76953125 | 13.039 | 6 | -3.16695 |
| TARGET-30-PALNLU | X | 76940087 | 76972722 | 32.636 | 7 | -1.659 |
| TARGET-30-PANZVU | X | 76940500 | 76972722 | 32.223 | 6 | -2.7205 |

(c)

| **sample** | **chr** | **start** | **end** | **length(Kb)** | **CNV_type** | **copy_number_estimate** | **original cov** | **normalized cov** | **mBIC** | **log-likelihood** |
| --- | --- | --- | --- | --- | --- | --- | --- | --- | --- | --- |
| TARGET-30-PATGLU | 23 | 76763827 | 76829825 | 66 | del | 0.66 | 258 | 776.74 | 188.13 | 209.54 |
| TARGET-30-PARACS | 23 | 76778728 | 76972722 | 193.99 | del | 0.26 | 808 | 6303.49 | 14341.1 | 2591.68 |
| TARGET-30-PANLET | 23 | 76912048 | 76972722 | 60.67 | del | 0.18 | 322 | 3607.99 | 1250.91 | 1900.32 |
| TARGET-30-PALFPI | 23 | 76918869 | 76954119 | 35.25 | del | 0.3 | 482 | 3215.44 | 1252.09 | 1273.62 |
| TARGET-30-PARKNP | 23 | 76931719 | 76972722 | 41 | del | 0.44 | 909 | 4085.92 | 1391.41 | 1412.89 |
| TARGET-30-PASRFS | 23 | 76931719 | 76972722 | 41 | del | 1.07 | 3368 | 6280.15 | 784.08 | 822.27 |
| TARGET-30-PAPKXS | 23 | 76931719 | 76972722 | 41 | del | 0.19 | 442 | 4721.97 | 1247.82 | 2377.91 |
| TARGET-30-PAKZRF | 23 | 76931719 | 76972722 | 41 | del | 0.34 | 574 | 3426.45 | 4850.81 | 1315.36 |
| TARGET-30-PANZVU | 23 | 76931719 | 76972722 | 41 | del | 1.12 | 2133 | 3804.46 | 402.27 | 434.33 |
| TARGET-30-PARMLF | 23 | 76931719 | 76972722 | 41 | del | 0.49 | 758 | 3116.96 | 1011.6 | 1033.08 |
| TARGET-30-PASAAN | 23 | 76931719 | 76972722 | 41 | del | 1.13 | 1455 | 2568.99 | 254.49 | 283.19 |
| TARGET-30-PASWLY | 23 | 76931719 | 76972722 | 41 | del | 0.71 | 566 | 1587.82 | 380.11 | 401.59 |
| TARGET-30-PAIFXV | 23 | 76937010 | 76952194 | 15.18 | del | 1.21 | 3304 | 5440.3 | 1694.79 | 446.39 |
| TARGET-30-PANXJL | 23 | 76937010 | 76954119 | 17.11 | del | 0.88 | 1320 | 2991.87 | 559.65 | 580.98 |
| TARGET-30-PAILNU | 23 | 76937010 | 76954119 | 17.11 | del | 1.09 | 4512 | 8305.7 | 1004.04 | 1047.76 |
| TARGET-30-PAMVLG | 23 | 76937010 | 76972722 | 35.71 | del | 0.21 | 418 | 3993.29 | 756.75 | 1776.51 |
| TARGET-30-PALNLU | 23 | 76937010 | 76972722 | 35.71 | del | 0.79 | 2762 | 6954.8 | 1541.52 | 1562.93 |
| TARGET-30-PASTCN | 23 | 76937010 | 76972722 | 35.71 | del | 0.26 | 574 | 4365.7 | 8141.52 | 1784.98 |

(d)

| **SAMPLE** | **CNV** | **INTERVAL** | **KB** | **CHR** | **MID_BP** | **TARGETS** | **NUM_TARG** | **Q_EXACT** | **Q_SOME** | **Q_NON_DIPLOID** | **Q_START** | **Q_STOP** | **MEAN_RD** | **MEAN_ORIG_RD** |
| --- | --- | --- | --- | --- | --- | --- | --- | --- | --- | --- | --- | --- | --- | --- |
| TARGET-30-PATGLU | DEL | X:76763827-76829825 | 66 | X | 76796826 | 2197..2204 | 8 | 69 | 99 | 99 | 34 | 12 | -6.24 | 21.41 |
| TARGET-30-PARACS | DEL | X:76778728-76972722 | 194 | X | 76875725 | 2201..2230 | 30 | 26 | 99 | 99 | 20 | 23 | -3.04 | 10.77 |
| TARGET-30-PANLET | DEL | X:76912048-76972722 | 60.67 | X | 76942385 | 2219..2230 | 12 | 11 | 99 | 99 | 11 | 22 | -5.7 | 6.4 |
| TARGET-30-PAILNU | DEL | X:76931719-76954119 | 22.4 | X | 76942919 | 2222..2229 | 8 | 7 | 99 | 99 | 7 | 8 | -4.75 | 123.68 |
| TARGET-30-PARKNP | DEL | X:76931719-76972722 | 41 | X | 76952220 | 2222..2230 | 9 | 36 | 99 | 99 | 16 | 33 | -6.74 | 17.76 |
| TARGET-30-PAPKXS | DEL | X:76931719-76972722 | 41 | X | 76952220 | 2222..2230 | 9 | 35 | 99 | 99 | 22 | 19 | -7.21 | 9.64 |
| TARGET-30-PASWLY | DEL | X:76931719-76972722 | 41 | X | 76952220 | 2222..2230 | 9 | 3 | 63 | 63 | 4 | 20 | -2.78 | 13.47 |
| TARGET-30-PARMLF | DEL | X:76931719-76972722 | 41 | X | 76952220 | 2222..2230 | 9 | 25 | 99 | 99 | 24 | 25 | -5.86 | 16.15 |
| TARGET-30-PASAAN | DEL | X:76931719-76972722 | 41 | X | 76952220 | 2222..2230 | 9 | 6 | 99 | 99 | 6 | 34 | -3.45 | 27.47 |
| TARGET-30-PALNLU | DEL | X:76931719-76972722 | 41 | X | 76952220 | 2222..2230 | 9 | 15 | 99 | 99 | 8 | 26 | -4.9 | 62.63 |
| TARGET-30-PANXJL | DEL | X:76937010-76954119 | 17.11 | X | 76945564 | 2223..2229 | 7 | 23 | 99 | 99 | 6 | 3 | -5 | 23.68 |
| TARGET-30-PASTCN | DEL | X:76937010-76972722 | 35.71 | X | 76954866 | 2223..2230 | 8 | 27 | 99 | 99 | 21 | 25 | -3.3 | 11.88 |
| TARGET-30-PAMVLG | DEL | X:76937010-76972722 | 35.71 | X | 76954866 | 2223..2230 | 8 | 96 | 99 | 99 | 23 | 32 | -6.65 | 8.39 |
| TARGET-30-PANZVU | DEL | X:76937010-76972722 | 35.71 | X | 76954866 | 2223..2230 | 8 | 13 | 99 | 99 | 4 | 31 | -3.67 | 24.23 |

**Supplementary Table S6. Genome-wide CNVs detected by CODEX of the neuroblastoma data set.** Blood and tumor CNVs are reported separately by chromosome.

| **Chr** | **Number of Targets** | **Optimal K** | **Blood CNVs (common-rare)** | **Tumor CNVs (common-rare)** |
| --- | --- | --- | --- | --- |
| 1 | 19157 | 4 | 916 (448-468) | 1303 (717-586) |
| 2 | 14033 | 4 | 386 (115-271) | 866 (360-506) |
| 3 | 11257 | 4 | 213 (0-213) | 321 (120-201) |
| 4 | 7365 | 3 | 232 (98-134) | 552 (295-257) |
| 5 | 8472 | 3 | 384 (180-204) | 755 (400-355) |
| 6 | 9346 | 4 | 421 (234-187) | 431 (239-192) |
| 7 | 8694 | 4 | 625 (436-189) | 775 (513-262) |
| 8 | 6353 | 3 | 267 (106-161) | 418 (179-239) |
| 9 | 7523 | 3 | 185 (55-130) | 307 (163-144) |
| 10 | 7730 | 3 | 241 (80-161) | 329 (182-147) |
| 11 | 10564 | 4 | 648 (147-501) | 1562 (822-740) |
| 12 | 10647 | 4 | 308 (34-274) | 464 (113-351) |
| 13 | 3332 | 3 | 101 (13-88) | 116 (20-96) |
| 14 | 5676 | 4 | 159 (41-118) | 173 (51-122) |
| 15 | 6479 | 3 | 158 (45-113) | 361 (137-224) |
| 16 | 7785 | 4 | 408 (221-187) | 488 (251-237) |
| 17 | 11208 | 4 | 304 (138-166) | 761 (500-261) |
| 18 | 2807 | 3 | 45 (0-45) | 84 (2-82) |
| 19 | 10780 | 5 | 558 (316-242) | 833 (377-456) |
| 20 | 4650 | 3 | 97 (30-67) | 108 (26-82) |
| 21 | 1889 | 3 | 72 (24-48) | 77 (39-38) |
| 22 | 3929 | 3 | 116 (51-65) | 146 (59-87) |
| X | 6445 | 3 | 157 (45-112) | 214 (74-140) |
| Y | 306 | 2 | 0 (0-0) | 0 (0-0) |
| All | 186427 | - | 7001 (2857-4144) | 11444 (5639-5805) |
